# Identification and Validation of a Multigene Predictor of Recurrence in Primary Laryngeal Cancer

**DOI:** 10.1371/journal.pone.0070429

**Published:** 2013-08-09

**Authors:** Elena Fountzilas, Vassiliki Kotoula, Nikolaos Angouridakis, Ilias Karasmanis, Ralph M. Wirtz, Anastasia G. Eleftheraki, Elke Veltrup, Konstantinos Markou, Angelos Nikolaou, Dimitrios Pectasides, George Fountzilas

**Affiliations:** 1 Department of Medical Oncology, “Papageorgiou” Hospital, Aristotle University of Thessaloniki School of Medicine, Thessaloniki, Greece; 2 Department of Pathology, Aristotle University of Thessaloniki School of Medicine, Thessaloniki, Greece; 3 ENT Department, “AHEPA” Hospital, Aristotle University of Thessaloniki School of Medicine, Thessaloniki, Greece; 4 Siemens Healthcare Diagnostics, Cologne, Germany; 5 Section of Biostatistics, Hellenic Cooperative Oncology Group, Data Office, Athens, Greece; 6 Oncology Section, Second Department of Internal Medicine, “Hippokration” Hospital, University of Athens School of Medicine, Athens, Greece; Univesity of Texas Southwestern Medical Center at Dallas, United States of America

## Abstract

**Purpose:**

Local recurrence is the major manifestation of treatment failure in patients with operable laryngeal carcinoma. Established clinicopathological factors cannot sufficiently predict patients that are likely to recur after treatment. Additional tools are therefore required to accurately identify patients at high risk for recurrence. This study attempts to identify and independently validate gene expression models, prognostic of disease-free survival (DFS) in operable laryngeal cancer.

**Materials and Methods:**

Using Affymetrix U133A Genechips, we profiled fresh-frozen tumor tissues from 66 patients with laryngeal cancer treated locally with surgery. We applied Cox regression proportional hazards modeling to identify multigene predictors of recurrence. Gene models were then validated in two independent cohorts of 54 and 187 patients (fresh-frozen and formalin-fixed tissue validation sets, respectively).

**Results:**

We focused on genes univariately associated with DFS (*p*<0.01) in the training set. Among several models comprising different numbers of genes, a 30-probe set model demonstrated optimal performance in both the training (log-rank, *p*<0.001) and 1^st^ validation (*p* = 0.010) sets. Specifically, in the 1^st^ validation set, median DFS as predicted by the 30-probe set model, was 34 and 80 months for high- and low-risk patients, respectively. Hazard ratio (HR) for recurrence in the high-risk group was 3.87 (95% CI 1.28–11.73, Wald's *p* = 0.017). Testing the expression of selected genes from the above model in the 2^nd^ validation set, with qPCR, revealed significant associations of single markers, such as ACE2, FLOT1 and PRKD1, with patient DFS. High PRKD1 remained an unfavorable prognostic marker upon multivariate analysis (HR = 2.00, 95% CI 1.28–3.14, *p* = 0.002) along with positive nodal status.

**Conclusions:**

We have established and validated gene models that can successfully stratify patients with laryngeal cancer, based on their risk for recurrence. It seems worthy to prospectively validate PRKD1 expression as a laryngeal cancer prognostic marker, for routine clinical applications.

## Introduction

Laryngeal cancer is the eleventh most common type of cancer in men worldwide. Every year 52,000 cases are newly diagnosed in Europe and 10,000 in the United States [1], [2]. Despite the latest advances in diagnostic and therapeutic techniques, the majority of patients still recur after treatment [3]. Established clinicopathological factors cannot sufficiently predict patients that will recur. Additional factors are therefore required to accurately identify patients with poor prognosis. Expression profiling has been successfully used in the stratification of cancer patients with unfavorable prognosis [4], [5], [6], [7]. Previous studies in head and neck cancer patients have linked gene expression profiles to nodal status [8], [9], [10], distant metastases [11], [12] and disease-free survival [13], [14], [15], [16]. While these studies provided great insight into the molecular complexity of head and neck cancer they did not identify a robust gene profile. The clinical use of these models has been limited by the large number of genes, the small-sized datasets and the lack of reproducibility and independent validation. Moreover, none of these studies focused exclusively on laryngeal cancer.

In the present study, we sought to identify genes prognostic of recurrence in patients with primary laryngeal cancer. The end-point of our analyses was disease-free survival (DFS). We profiled tumor samples from two separate cohorts of patients using global gene expression profiling. Using the first cohort as a training set, we identified several prognostic gene models, which were then validated in the second cohort of patients. In order to further validate our results, we profiled selected genes of our models for relative expression with quantitative real time-polymerase chain reaction (qRT-PCR) in an independent cohort of patients.

## Materials and Methods

### Study population

Our study comprised of 307 patients diagnosed with primary squamous cell laryngeal cancinoma (training set: 66, 1^st^ validation set: 54 and 2^nd^ validation set: 187 patients), with median follow-up of 52, 33 and 89 months, respectively. All patients underwent surgical removal of the tumor at the Otorhinolaryngology Department of the AHEPA Hospital in Thessaloniki, Greece, between 1985 and 2008. Postoperative administration of radiation therapy was decided by the treating physician and most often was given to patients with positive tumor margins, patients with T4 tumors or those with T1/T2 supraglottic tumors for whom a prophylactic elective lymphadenectomy was not done. None of the patients received systemic therapy as part of their initial treatment. This is the current standard of care in many European countries [17], however this may not be the case in other countries, like the US, where the main focus is the preservation of the larynx with concurrent chemoradiotherapy, preceded in select cases by induction chemotherapy [18]. Follow-up included physical examination, every three months for the first three years and every six months thereafter. Imaging examinations were performed, as indicated by symptoms and physical examination. Detailed demographics and clinical characteristics for the patients with valid gene data are listed in Table 1, while individual patient data are shown as in Table S1.

**Table 1:** Patient Demographics and Clinical Characteristics for the Training and Validation Sets.

	Training Set	1^st^ Validation Set	2^nd^ Validation Set	*P*-value*	*P*-value**
	**N = 59**	**N = 50**	**N = 149**		
**Age**					
Median (range)	62 (41–88)	64 (41–82)	62 (42–80)	0.245	0.877
	**n (%)**	**n (%)**	**n (%)**		
**Gender**				0.372	0.276
Female	4 (6.8)	1 (2.0)	5 (3.4)		
Male	55 (93.2)	49 (98.0)	144 (96.6)		
**Smoking**				1.000	0.450
No	1 (1.7)	0 (0)	8 (5.4)		
Yes	58 (98.3)	52 (100.0)	141 (94.6)		
**Alcohol**				0.341	0.127
No/Mild	25 (42.4)	26 (52.0)	81 (54.4)		
Moderate/Heavy	34 (57.6)	24 (48.0)	68 (45.6)		
**Stage**				0.009	0.260
1	1 (1.7)	11 (22.0)	9 (6.0)		
2	10 (16.9)	8 (16.0)	19 (12.8)		
3	22 (37.3)	14 (28.0)	66 (44.3)		
4	26 (44.1)	17 (34.0)	49 (32.9)		
Unknown	-	-	6 (4.0)		
**Grade**				0.381	0.778
1	19 (32.2)	23 (46.0)	53 (35.6)		
2	28 (47.5)	21 (42.0)	61 (40.9)		
3	10 (16.9)	6 (12.0)	25 (16.8)		
Unknown	2 (3.4)	-	10 (6.7)		
**Radiation therapy**				0.54	0.006
No	23 (39.0)	20 (40.0)	105 (70.5)		
Yes	25 (42.4)	29 (58.0)	44 (29.5)		
Unknown	11 (18.6)	1 (2.0)	0 (0.0)		
**Recurrence**				0.037	<0.001
No	46 (78.0)	29 (58.0)	65 (43.6)		
Yes	13 (22.0)	21 (42.0)	84 (56.4)		

* denotes comparisons between Training vs 1^st^ Validation set.

** denotes comparison between Training vs 2^nd^ Validation set.

### Tumor specimens

Fresh-frozen tumor tissue samples, from patients comprising the training and 1^st^ validation sets, were prospectively collected at the time of surgery, from 2004 to 2008, were immediately frozen in liquid nitrogen and stored in −80°C until processing. Formalin-fixed paraffin-embedded (FFPE) tumor tissue samples, from patients comprising the 2^nd^ validation set, were retrospectively collected (patients treated between 1985 and 2008). The latter were fixed in formalin for at least 6 hours before being embedded in paraffin. Laryngeal tumors were histologically assessed and verified in all cases, including the fresh-frozen tissue samples.

### Ethics Statement

Fresh-frozen and FFPE tumor tissue samples were collected according to protocols approved by the Institutional Review Board of the “AHEPA” Hospital and the Bioethics Committee of the Aristotle University of Thessaloniki, School of Medicine. Written informed consent for the scientific use of biological material was obtained from all patients comprising the training and 1^st^ validation sets and from the patients of the 2^nd^ validation set treated after 2004. Waiver of consent was obtained from the Bioethics Committee for patients treated before 2004 and for whom FFPE tumor tissue samples needed to be retrospectively collected. All clinical investigations related to the present study have been conducted according to the principles expressed in the Declaration of Helsinki.

### RNA isolation from fresh-frozen tissue and global gene expression profiling

RNA isolation from fresh frozen tumor specimens was performed using the RNeasy protocol (Qiagen, Hilden, Germany), as previously described [19]. RNA quantity was determined by measuring UV absorbance at 260 and 280 nm, while RNA quality was assessed using an Agilent 2100 Bioanalyzer RNA 6000 LabChip kit (Agilent Technologies, Palo Alto, CA). RNA was reverse transcribed, labeled and hybridized to Affymetrix (Santa Clara, CA) HG-U133A arrays, as previously described [19]. Experiments concerning the training and 1^st^ validation sets were carried out separately, at different time points, at Siemens Healthcare Diagnostic Products (Cologne, Germany). The gene expression data have been deposited in the National Center for Biotechnology Information Gene Expression Omnibus (GEO, http://www.ncbi.nlm.nih.gov/geo/) and are available through the GEO Series accession number GSE27020. The following link has been created to allow review of GSE27020: http://www.ncbi.nlm.nih.gov/geo/query/acc.cgi?token=dxcdxksauqicizy&acc=GSE27020

### RNA isolation from FFPE tumor tissue samples and qRT-PCR

All investigations involving FFPE tissue samples were performed at the Laboratory of Molecular Oncology, Hellenic Foundation for Cancer Research, Aristotle University of Thessaloniki School of Medicine. To validate the prognostic value of genes derived from our microarray analysis, we used a separate cohort of patients with available tumor tissue material. This sample group included 187 FFPE tissue samples that were macrodissected upon previous histological evaluation to contain >50% tumor cells. RNA was isolated after complete overnight tissue lysis using Trizol-LS (Life Technologies, Paisley, UK), as previously described [20] and was reverse transcribed with Superscript III (Life Technologies), according to the manufacturer's instructions. cDNAs were normalized at 25 ng/ul and stored at −20°C until use. mRNA expression was investigated with FAM-labeled TaqMan® Gene Expression Assays in duplex reactions involving a primer-limited VIC-labeled reference assay for glucuronidase beta (GUSB, assay # Hs00939627_m1), as an endogenous template control. GUSB was preferred over usually applied endogenous controls because no pseudogenes have, as yet, been reported for this gene. Additionally, it has been identified as one among the best preserved mRNA targets in FFPE tissues [21]. qPCR mRNA target selection included evaluation of the 30 probes of the U133A signature for gene duplicates, gene characteristics (valid gene identities, type of gene, recorded splice variants that would not be distinguished by the probe on the array or by the qPCR assay), as well as a parametric *p*-value of <0.05 for fold-change in gene expression. Out of the 30 U133A probes, 23 remained valid for qRT-PCR application (Table 2). For these 23 targets, we searched for pre-made TaqMan® Gene Expression Assays (Applied Biosystems) that would match the target sequences detected by the corresponding probes in the U133A array. It was possible to retrieve 16 such assays (Table 2). Duplex 10 ul reactions were run in duplicates, each containing 50 ng of template, in an ABI PRISM 7900HT system (Applied Biosystems/Life Technologies) on a 384-well block under default conditions involving 45 amplification cycles, along with a reference RNA sample (TaqMan® Control Total RNA, cat. no 4307281, Applied Biosystems) and no-template controls. The reference RNA was used as a positive plate control and for the evaluation of assay performance among runs (inter-run validation). Two of the 16 selected assays yielded deltaRQ values of >1.5 among different runs and, hence, failed inter-run validation and were not further evaluated (Table 2).

**Table 2:** mRNA target selection from the 30 probes included in the prognostically relevant signature and TaqMan® gene expression assay description.

Order	U133A probe set	Gene name	GB accession (U133A robe)	Gene symbol	Gene ID	Gene characteristics	Parametric p-value	Fold change	qPCR assay employed	Reason for exclusion	GB Reference (qPCR assay)	Assay ID*	Amplicon size	Exon spanning	Accepted/Failed
1	219962_at	Angiotensin I converting enzyme (peptidyl-dipeptidase A) 2	NM_021804	ACE2	59272		0,0660013	1,06	yes		NM_021804.2	Hs01085334_m1	83	18–19	**Accepted**
2	209916_at	Dehydrogenase E1 and transketolase domain containing 1	BC002477	DHTKD1	55526		0,0022442	1,12	yes		NM_018706.5	Hs00907842_g1	82	16–17	**Accepted**
3	208748_s_at	Flotillin 1	AA507012	FLOT1	10211		0,1379991	0,96	yes		NM_005803.2	Hs01561949_g1	84	3–4	**Accepted**
4	214219_x_at	Mitogen-activated protein kinase kinase kinase kinase 1	BE646618	MAP4K1	11184	splice variants	<1e-07	1,27	yes		NM_001042600.1 NM_007181.4	Hs01018257_g1	66	28–29	**Accepted**
5	211080_s_at	NIMA (never in mitosis gene a)-related kinase 2	Z25425	NEK2	4751	splice variants	0,0133109	0,94	yes		NM_002497.2	Hs00601227_mH	124	4–5	**Accepted**
6	205880_at	Protein kinase D1	NM_002742	PRKD1	5587		0,0485631	0,95	yes		NM_002742.2	Hs01554316_m1	65	13–14	**Accepted**
7	202773_s_a	Splicing factor, arginine/serine-rich 8 (suppressor-of-white-apricot homolog, Drosophila)	AI023864	SFRS8	6433	splice variants	0,0068728	1,08	yes		NM_004592.2	Hs00902642_m1	60	4–5	**Accepted**
8	203387_s_at	TBC1 domain family, member 4	NM_014832	TBC1D4	9882		0,0001543	1,24	yes		NM_014832.2	Hs00952770_m1	134	20–21	**Accepted**
9	203834_s_at	Trans-golgi network protein 2	NM_006464	TGOLN2	10618	splice variants	6,38E-05	0,89	yes		NM_006464.2	Hs00197728_m1	79	2–3	**Accepted**
10	205836_s_at	YTH domain containing 2	NM_022828	YTHDC2	64848		0,0981884	1,04	yes		NM_022828.3	Hs00967276_g1	75	29–30	**Accepted**
11	219719_at	HIG1 domain family, member 1B	NM_016438	HIGD1B	51751		0,8940065	1	yes		NM_016438.2	Hs00212725_m1	117	2–3	Failed ∧(1)
12	219464_at	Carbonic anhydrase XIV	NM_012113	CA14	23632		9,00E-07	1,17	yes		NM_012113.1	Hs00201626_m1	66	10–11	Failed #
13	205303_at	Potassium inwardly-rectifying channel, subfamily J, member 8	BF514158	KCNJ8	3764		0,2259278	0,97	yes		NM_004982.2	Hs00958961_m1	88	2–3	Failed ∧(1)
14	217543_s_at	Membrane-bound transcription factor peptidase, site 1	BE890314	MBTPS1	8720		0,0004541	0,87	yes		NM_003791.2	Hs00921633_m1	85	19–20	Failed ∧(1)
15	215616_s_at	Jumonji domain containing 2B	AB020683	JMJD2B	23030		0,0001684	1,08	yes		NM_015015.2	Hs00943636_m1	167	22–23	Failed ∧(2)
16	206617_s_at	Renin binding protein	NM_002910	RENBP	5973		4,54E-05	1,14	yes		NM_002910.5	Hs00234138_m1	75	9–10	Failed #
17	222257_s_at	Angiotensin I converting enzyme (peptidyl-dipeptidase A) 2	AK026461	ACE2	59272		7,38E-05	0,87	duplicate	higher p-value					
18	214339_s_at	Mitogen-activated protein kinase kinase kinase kinase 1	AA744529	MAP4K1	11184	splice variants	1,40E-06	1,18	duplicate	higher p-value					
19	216848_at		AB051447	cancelled (unknown transcriptional capacity)			8,00E-07	1,15	no	irrelevant					
20	214561_at	Leukocyte immunoglobulin-like receptor pseudogene 2	NM_024317	LILRP2	79166	pseudogene	0,0025841	1,08	no	pseudogene					
21	210257_x_at	Cullin 4B	AF212995	CUL4B	8450	splice variants	1,53E-05	1,21	no	no matching assay					
22	222090_at	Hypothetical protein LOC100134713	BF509069	LOC100134713	100134713	antisense RNA	4,50E-05	1,09	no	no matching assay					
23	213615_at	Lysophosphatidylcholine acyltransferase 3	AA773554	LPCAT3	10162		6,00E-07	1,12	no	no matching assay					
24	213906_at	v-myb myeloblastosis viral oncogene homolog (avian)-like 1	AW592266	MYBL1	4603	splice variants	0,001513	0,89	no	no matching assay					
25	214885_at	MYST histone acetyltransferase 1	AL050395	MYST1	84148	splice variants	2,30E-06	1,17	no	no matching assay					
26	213590_at	Solute carrier family 16, member 5 (monocarboxylic acid transporter 6)	AA705628	SLC16A5	9121		4,89E-05	1,18	no	no matching assay					
27	219103_at	ArfGAP with SH3 domain, ankyrin repeat and PH domain 3	NM_017707	ASAP3	55616	splice variants	8,20E-06	1,11	no	no matching assay					
28	38043_at	Family with sequence similarity 3, member A	X55448	FAM3A	60343	splice variants	0,1878481	0,96	no	splice variants, not significant p					
29	206194_at	Homeobox C6	AW299598	HOXC6	3223	splice variants	0,0207144	0,94	no	splice variants, not significant p					
30	215371_at	Mediator complex subunit 27	AU147599	MED27	9442	splice variants	0,0815968	1,04	no	splice variants, not significant p					

* Applied Biosystems/Life Technologies.

# High inter-run variations of the RQ values for the reference RNA sample (inter-run deltaRQ value range for the reference RNA (19 runs) and for 23 samples (monthly interval between runs) >1.5.

∧(1) Complete absence of amplification curves in the reference RNA and/or in all study samples.

∧(2) High intra-run variability in deltaRQ values for study duplicates with amplified templates (duplicate deltaRQ >0.8).

Cycle thresholds (CT, corresponding to Cq in MIQE guidelines) for each target and for the endogenous reference were automatically obtained at default conditions and relative quantification (RQ) was calculated in a linear mode [22] by subtracting (45–avg deltaCT), whereby 45 was the total amplification cycle number and avg dCT = average [(CT target)−(CT GUSB)] for duplicates. Eligibility criteria for further sample evaluation included GUSB CT values of <38 for each reaction and deltaRQ for each duplicate (intra-run variation) of <0.8. For 3 assays no amplification curves were obtained for the FFPE and the reference mRNA samples, while for an additional assay high intra-run RQ value differences were observed in 87% of samples (Table 2). Based on the above filtering steps for assay and sample eligibility, it was finally possible to evaluate RQ results for 10 genes only.

By applying the above criteria for sample eligibility, the following number of informative samples was obtained per mRNA target (informative assays only): ACE2, 159; DHTKD1 171; FLOT1, 178; MAP4K1, 169; NEK2, 156; SFRS8, 184; PRKD1, 162; TBC1D4, 165; TGOLN2, 183 and YTHDC2, 176.

### Statistical analysis

Prognostic gene expression models were developed exclusively in the training set. DFS was measured from the time of diagnosis until verified disease progression or death. Alive patients without verified disease progression were censored at the date of last contact. Genes selected had to be univariately associated with DFS (*p*<0.01, Cox proportional hazard model). The algorithm fits proportional hazards models to relate DFS to each gene, one gene at a time, and provides a *p* value for each gene, testing the hypothesis that DFS is independent of the expression level of the particular gene. Genes found to be associated with DFS in the training set were then ranked based on their absolute hazard ratio value, provided by the algorithm. Prognostic gene models, comprising different numbers of top ranking genes, were developed using the supervised principal component survival algorithm [23]. The algorithm computes principal components and performs Cox proportional hazard regression analysis to calculate a regression coefficient (weight) for each principal component. A supervised principal component model is developed to provide a prognostic index for each patient of the study. A high prognostic index corresponds to a high value of hazard of recurrence. To evaluate the predictive value of this method, we used Leave-One-Out-Cross-Validation, where each case is omitted and the entire analysis is performed using the rest of the samples. In order to directly apply these models to the 1^st^ validation set, we normalized the training and the 1^st^ validation sets, using the empirical Bayes (EB) method [24]. The method uses an algorithm designed to adjust for the non-biological experimental variation (“batch effect”) between different datasets. It reduces inter-laboratory variation, as well as technical differences due to the utilization of different platforms and methodological approaches. After normalization, we directly applied the gene models to the 1^st^ validation set without any modifications.

Kaplan-Meier curves and log-rank tests were used to estimate and compare the survival distributions in patients at high- and low-risk of recurrence. All reported *p* values are two-sided. Cox proportional hazard analysis was used for univariate analysis and multivariate adjustment for known prognostic factors. Statistical analysis was performed using the BRB-ArrayTools developed by Dr. Richard Simon and the BRB-ArrayTools Development Team and the SPSS statistical package, version 18.0, (IBM Corporation, Armonk, NY).

We used the unsupervised “Subclass Mapping” (Submap) method [25] to evaluate the molecular correspondence of patients with favorable and unfavorable prognosis between the training set and the 1^st^ validation set. This method bi-directionally assesses the association of predefined subtypes in multiple independent datasets, despite their technical variation. The algorithm provides the calculation of a *p* value to demonstrate the likelihood of molecular similarity between the different subclasses, it is implemented in the GenePattern software (Version 3.0, Broad Institute, Cambridge, MA) and can be accessed at http://www.broad.mit.edu/genepattern/

Gene set analysis (GSA) was utilized to detect gene network deregulation characteristic of groups of patients with good or poor prognosis [26]. Using publicly available data, we then predicted oncogenic pathway activation status in each patient of the training and 1^st^ validation sets. We applied gene expression models, previously developed and validated in vitro, to estimate the probability of pathway activation in each sample [27]. Finally, using Bayesian probit regression models we assigned to each patient a probability of pathway activation.

## Results

### Identification and validation of prognostic classifiers using gene expression profiling

**Figure 1:**
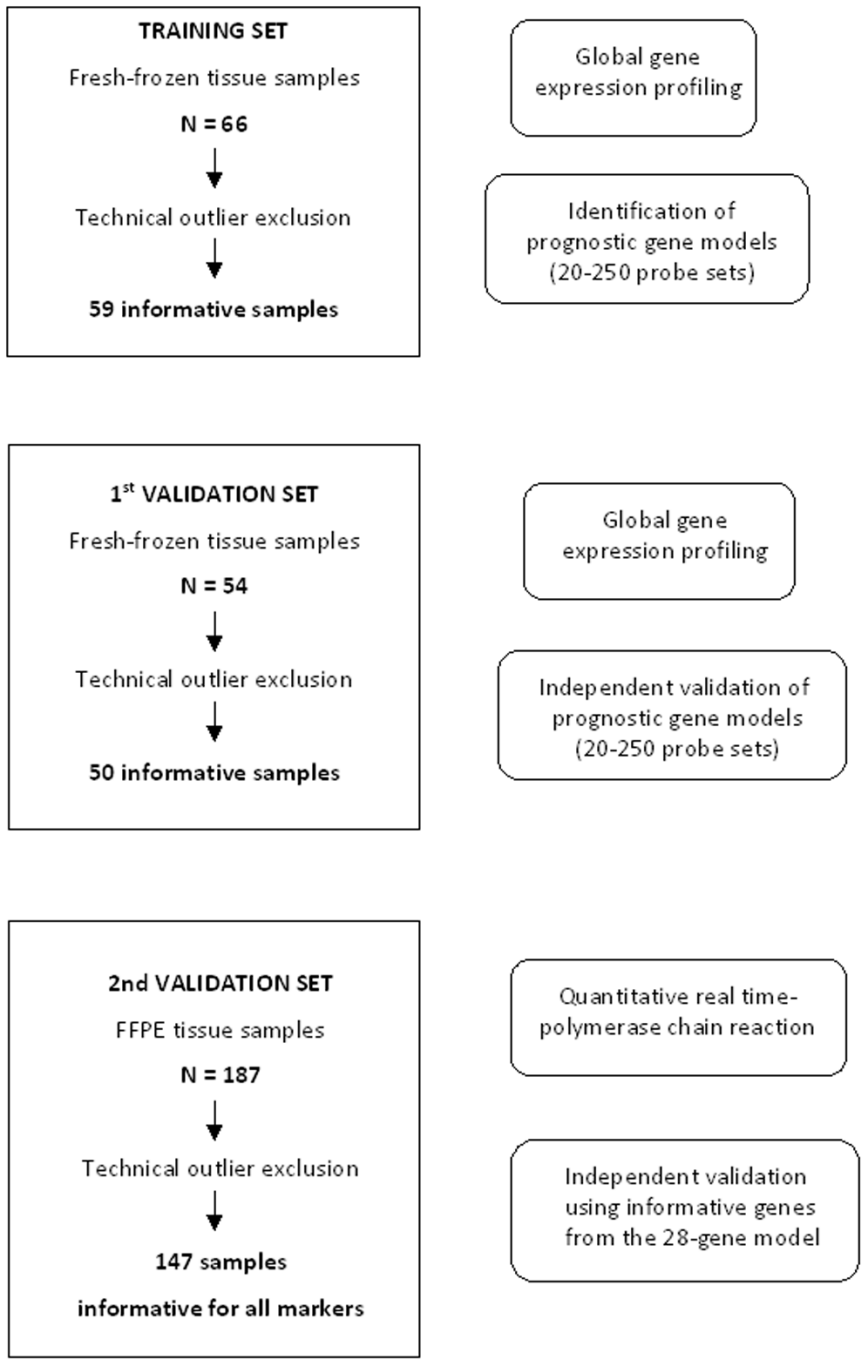
Consort diagram.

The flowchart of our study is shown in Figure 1 (consort diagram). We analyzed primary laryngeal tumors from 66 patients (training set) and 54 patients (1^st^ validation set) using global gene expression profiling. After evaluating the quality of the microarray data, we excluded 7 and 4 technical outliers from the two sets, respectively. For some of the genes, expression was evaluated using two different probe sets. Prognostic probe set models were identified exclusively in the training set. After excluding one fourth of the least variant genes, we focused on genes associated with DFS (Wald's *p*<0.01). We then ranked the 253 probe sets found to be significantly associated with DFS, based on their Cox regression coefficient. We identified several prognostic probe set models consisting of as many as 250 to as few as 20 probe sets, which performed equally well in the training set.

Subsequently, we applied these multigene predictors directly to the 1^st^ validation set. The 30-probe set model performed the best in the validation set (the model with the fewest genes demonstrating the highest statistical difference in DFS between high- and low-risk patients). Median DFS for the groups of patients with unfavorable and favorable prognosis, as predicted by the 30-probe set model, was 34 and 80 months, respectively (log-rank, *p* = 0.010). The hazard ratio (HR) for recurrence in the high-risk group versus the low-risk group was 3.87 (95% CI 1.28–11.73, Wald's *p* = 0.017). Kaplan-Meier curves for all probe set models in the training and 1^st^ validation sets can be found in File S1. Concordance between the risk assignments both in the training and 1^st^ validation sets based on the different classifiers was high, 81–87% (Cramer V test = 0.62 to 0.75).

**Figure 2:**
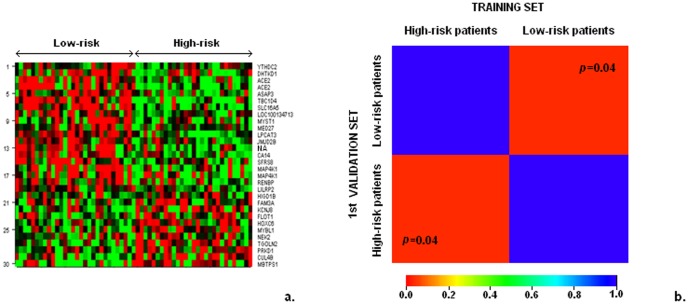
Graphical representation of the 30-probe set model expression patterns (a) and Submap results (b). Graphical representation of the 30-probe set model expression patterns in patients with favorable and unfavorable prognosis is shown in panel a. Red color denotes overexpression and green color underexpression of the respective genes. Molecular similarity of patients with favorable and unfavorable prognosis in the training and 1^st^ validation sets, using Submap, is shown in panel b. The bar below indicates the relationship between color and Bonferonni corrected *p* values. Red color represents high confidence for molecular homogeneity between the respective groups, while blue color represents lack of confidence thereof.

**Figure 3:**
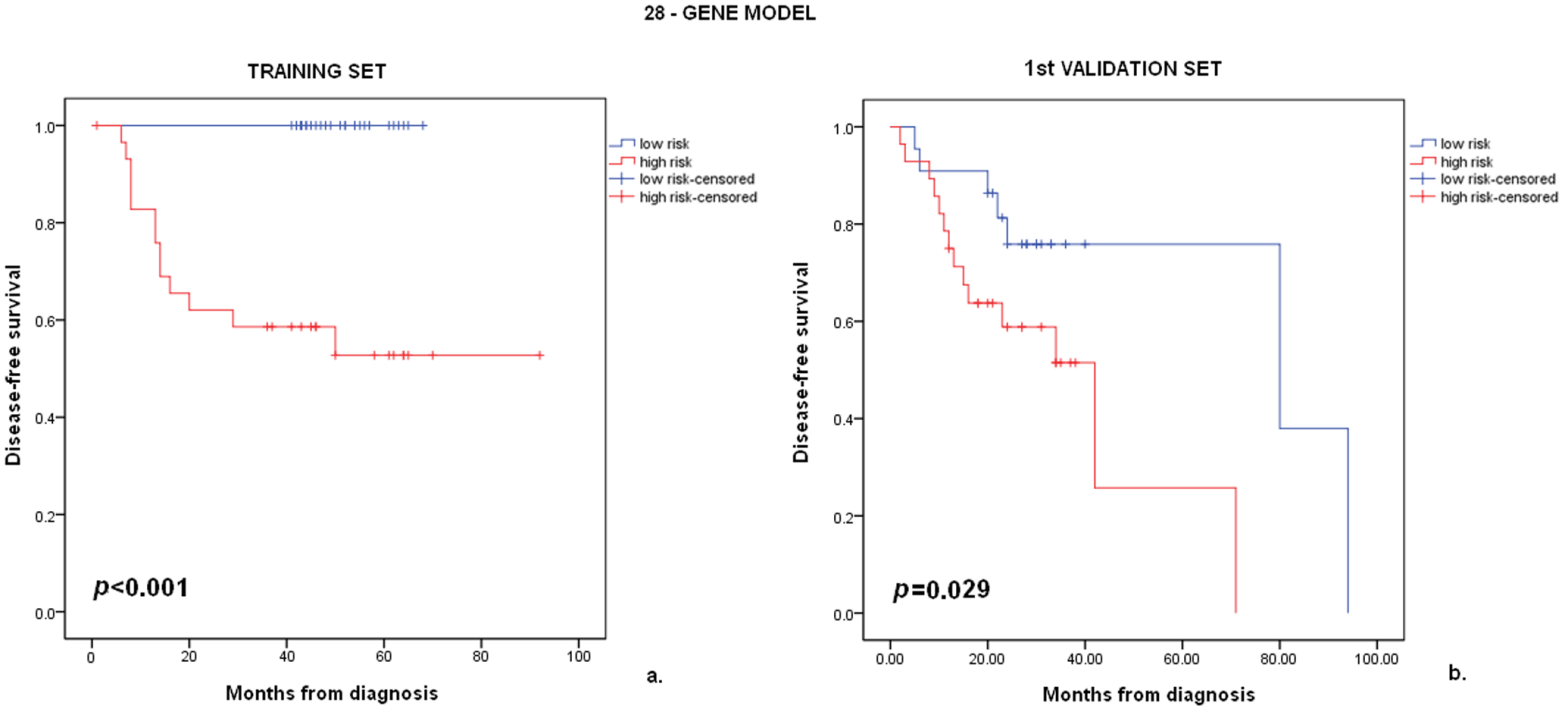
Kaplan-Meier survival estimates based on the 28-gene model risk predictions. Kaplan-Meier survival estimates for high- and low-risk patients based on the 28-gene model risk predictions in the training set (a) and 1^st^ validation set (b).

Annotations of all 30 probe sets included in our model are shown in Table 3, while a graphical representation of the gene expression patterns of the 30-probe set model in the high- and low-risk patients is shown in Figure 2a. We observed that in this model, two genes (ACE2 and MAP4K1) were represented by two probe sets. In order to avoid the effect of weighting these two genes twice as much compared to each of the other individual probe sets, representing a single gene, we retested our model in the training and 1^st^ validation sets using a single probe set for each gene. In the training set, statistical significance in 28-gene model remained identical (log-rank test, *p*<0.001, Figure 3a) with the one based on the 30-probe set model. Median DFS in the 1^st^ validation set, as predicted by the 28-gene model, was 34 and 80 months, respectively (log-rank, *p* = 0.029, Figure 3b). The hazard ratio (HR) for recurrence in the high-risk group versus the low-risk group was 4.38 (95% CI 1.06–7.01, Wald's *p* = 0.036).

**Table 3:** 30-probe set model annotations.

Probe set	Name	Symbol
202773_s_at	splicing factor, arginine/serine-rich 8 (suppressor-of-white-apricot homolog, Drosophila)	SFRS8
203387_s_at	TBC1 domain family, member 4	TBC1D4
203834_s_at	trans-golgi network protein 2	TGOLN2
205303_at	potassium inwardly-rectifying channel, subfamily J, member 8	KCNJ8
205836_s_at	YTH domain containing 2	YTHDC2
205880_at	protein kinase D1	PRKD1
206194_at	homeobox C6	HOXC6
206617_s_at	renin binding protein	RENBP
208748_s_at	flotillin 1	FLOT1
209916_at	dehydrogenase E1 and transketolase domain containing 1	DHTKD1
210257_x_at	cullin 4B	CUL4B
211080_s_at	NIMA (never in mitosis gene a)-related kinase 2	NEK2
213590_at	solute carrier family 16, member 5 (monocarboxylic acid transporter 6)	SLC16A5
213615_at	lysophosphatidylcholine acyltransferase 3	LPCAT3
213906_at	v-myb myeloblastosis viral oncogene homolog (avian)-like 1	MYBL1
214219_x_at	mitogen-activated protein kinase kinase kinase kinase 1	MAP4K1
214339_s_at	mitogen-activated protein kinase kinase kinase kinase 1	MAP4K1
214561_at	leukocyte immunoglobulin-like receptor pseudogene 2	LILRP2
214885_at	MYST histone acetyltransferase 1	MYST1
215371_at	mediator complex subunit 27	MED27
215616_s_at	jumonji domain containing 2B	JMJD2B
216848_at	NA	NA
217543_s_at	membrane-bound transcription factor peptidase, site 1	MBTPS1
219103_at	ArfGAP with SH3 domain, ankyrin repeat and PH domain 3	ASAP3
219464_at	carbonic anhydrase XIV	CA14
219719_at	HIG1 domain family, member 1B	HIGD1B
219962_at	angiotensin I converting enzyme (peptidyl-dipeptidase A) 2	ACE2
222090_at	hypothetical protein LOC100134713	LOC100134713
222257_s_at	angiotensin I converting enzyme (peptidyl-dipeptidase A) 2	ACE2
38043_at	family with sequence similarity 3, member A	FAM3A

To further validate the prognostic significance of these profiles, we applied our 28-gene model to a publicly available cohort of patients with early stage laryngeal cancer [28]. Due to technical variation between the two datasets, 23 out of the 28 genes of our model were used to stratify patients based on their risk for recurrence. Despite the technical and biologic limitations (different platforms, different number of genes, early vs all-stage disease) our model maintained its prognostic significance. Median DFS for the groups of patients with unfavorable and favorable prognosis, as predicted by the 23-gene model, was 118 and 161 months, respectively (log-rank, *p* = 0.011, Figure S1). The HR for recurrence in the high-risk group versus the low-risk group was 4.37 (95% CI 1.24–15.34, Wald's *p* = 0.022).

### Molecular homology of patients with favorable and unfavorable prognosis in the training and 1^st^ validation sets

Our 28-gene profile appears to not solely be a collection of prognostic genes but to actually capture the underlying biology of the tumors. To demonstrate the molecular homogeneity of high-risk tumors, we used subclass mapping (Submap), a method that assesses molecular similarity of predefined groups belonging to different datasets. We indeed illustrated that groups of patients with poor and good prognosis in the training set share the same biological patterns with the respective groups in the validation set, above and beyond the expression of specific genes. Charts displaying good “molecular match” of the high- and low-risk patients are shown in Figure 2b.

### Independent prognostic significance of the 28-gene model

We were interested in demonstrating the independent prognostic significance of our multigene predictor. We included in the analysis stage and grade, the only known prognostic factors, for which data were available for the patients of our study. We have also included radiation therapy, since it is known to significantly reduce local recurrence. In multivariate analysis, our 28-gene model maintained borderline prognostic significance in the 1^st^ validation set. HR for recurrence in the high-risk group was 2.67 (95% CI 0.99–7.22, Wald's *p* = 0.05) (details are shown in Table 4).

**Table 4:** Univariate and multivariate analyses.

		Univariate *p* value	Hazard Ratio (HR)	95% CI		Multivariate *p* value	Hazard Ratio (HR)	95% CI	
				Lower	Upper			Lower	Upper
	**Prognostic signature**	_*	_*	_*	_*	_*	_*	_*	_*
**Training set**	**Stage**	0.45	1.33	0.64	2.77	0.67	0.75	0.20	2.82
	**Grade**	0.31	1.49	0.69	3.22	0.66	1.24	0.48	3.18
	**Radiation therapy**	0.16	2.61	0.69	9.86	0.43	1.84	0.40	8.53
**1st validation set**	**Prognostic signature**	0.04	2.73	1.07	7.01	0.05	2.67	0.99	7.22
	**Stage**	0.89	0.97	0.64	1.48	0.91	0.97	0.56	1.67
	**Grade**	0.75	0.9	0.45	1.78	0.52	0.77	0.35	1.69
	**Radiation therapy**	0.25	1.82	0.65	5.07	0.36	1.69	0.55	5.17

* Coefficients did not converge and no models could be fitted.

### Pathway analysis in the high- and low-risk groups

**Figure 4:**
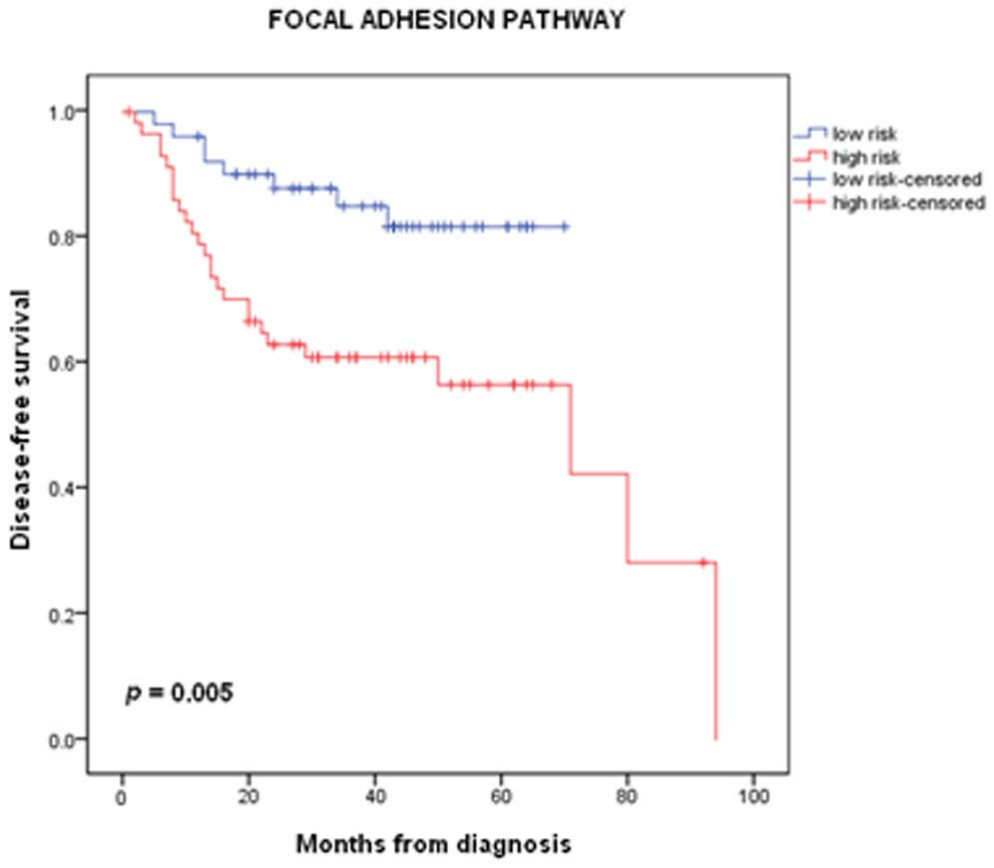
Focal Adhesion pathway. Kaplan-Meier disease-free survival estimates for high- and low-risk patients, as defined by the focal adhesion pathway genes (log-rank, *p*<0.005).

In order to gain some additional insight into the biological processes in patients with favorable and unfavorable prognosis, we performed Gene Set Analysis (GSA). We explored gene networks and biological themes, as described by the Kyoto Encyclopedia of Genes and Genomes (KEGG) Pathway Database. We indeed identified a wide range of pathways, differentially expressed between the two groups of patients (GSA Goeman's global test *p* values<0.01). We focused on pathways deregulated both in the training and in the 1^st^ validation sets. Table 5 presents selected pathways of interest, while the full list of pathways can be found as in Table S2. Several of these pathways have previously been shown to play an important role in head and neck cancer progression. Interestingly, we observed that genes of the focal adhesion (FA) pathway [29], shown to be prognostic in our dataset, as well as in head and neck cancer, successfully stratified our patients based on their risk of recurrence (details in Figure 4).

**Table 5:** Gene set analysis in patients with poor and good prognosis in the training and 1^st^ validation sets.

GENE SET ANALYSIS (GSA)	TRAINING SET	1^st^ VALIDATION SET
Pathways - Gene Networks	Goeman's global test *p*-value	Goeman's global test *p*-value
TGF-beta signaling pathway	0.0001	0.0003
VEGF signaling pathway	0.0002	0.0008
ECM-receptor interaction	0.0019	0.0009
Wnt signaling pathway	<0.0001	0.0011
mTOR signaling pathway	0.0012	0.0028
Hedgehog signaling pathway	0.0008	0.0031
Phosphatidylinositol signaling system	0.0044	0.0008
Insulin signaling pathway	<0.0001	0.0001
Focal adhesion	0.0008	0.0008
Nicotinate and nicotinamide metabolism	<0.0001	0.0002
Regulation of actin cytoskeleton	0.0001	0.0005

Selected pathways of interest, statistically significantly deregulated in patients with good prognosis compared to patients with poor prognosis, both in the training and 1^st^ validation sets are shown.

### Oncogenic pathway activation patterns in individual patients

**Figure 5:**
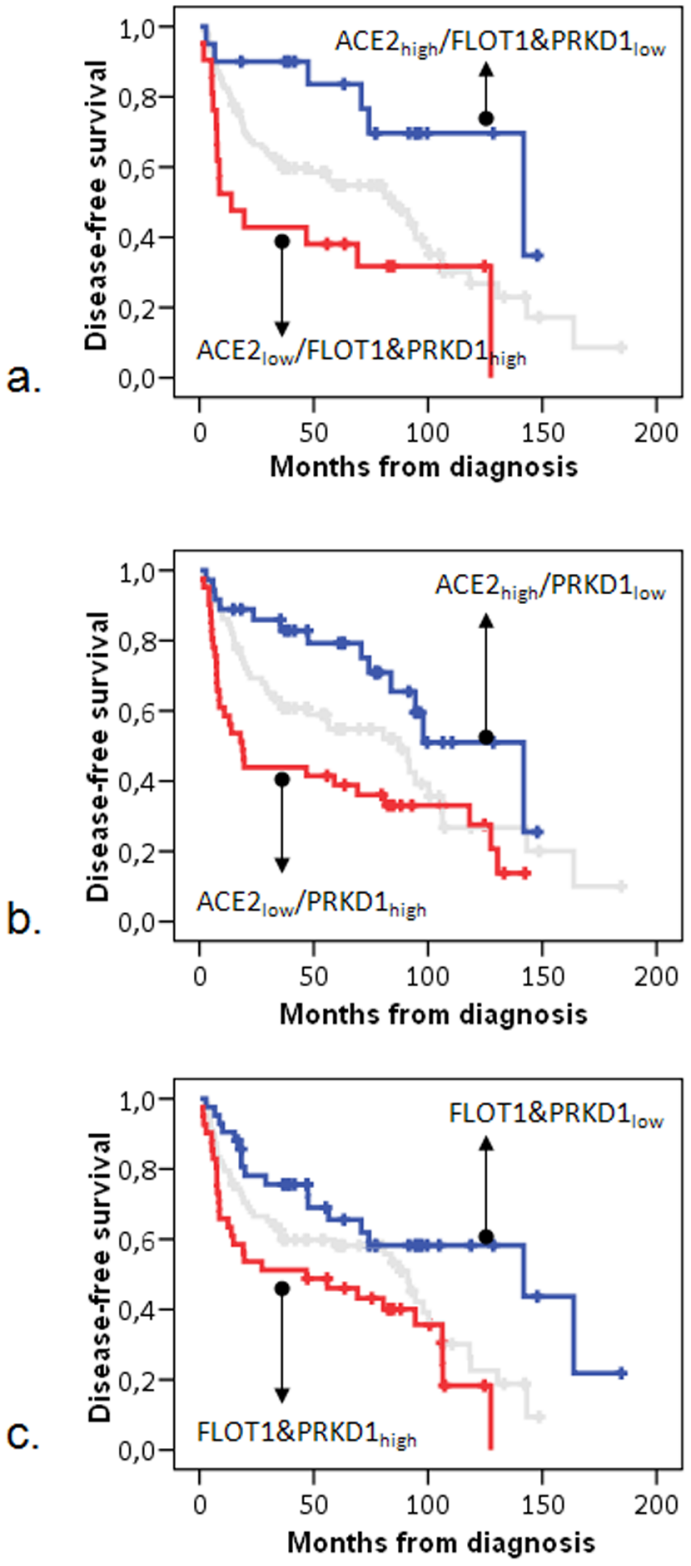
Kaplan-Meier curves showing the effect of combined ACE2, FLOT1 and PRKD1 expression on patient DFS. RQ values have been combined as binary variables to obtain 3-scale profiles according to array data. Markers as indicated. Blue and red curves match the expected prognostic patterns obtained in the 28-gene model for the particular genes. Grey curves: unclassified patients (intermediate scale).

In addition to the aforementioned pathway analysis results that derived from evaluating groups of patients, we sought to explore pathway activation in individual patients. We used previously developed and validated “in vitro” gene expression “read-outs” to identify activation of known oncogenic pathways in each patient of the training and 1^st^ validation sets. We investigated the Src, Ras, β-catenin and E2F3 pathways, which have previously been shown to be associated with survival in other types of cancer [27]. Interestingly, we demonstrated that patients with poor prognosis more often had tumors characterized by Ras pathway activation, odds ratio (OR) = 2.92 (95% CI 1.33–6.39, Wald's *p*<0.01), while patients with good prognosis more often had tumors exhibiting Src and β-catenin pathway activation, OR = 4.54 (95% CI 1.64–12.50, *p* = 0.003) and 2.63 (95% CI 1.16 to 5.88, *p* = 0.03), respectively. In addition, we performed Cox proportional hazards survival regression and observed that the Ras pathway activation was found to be associated with poor prognosis, HR = 2.55 (95% CI 1.19–5.45, *p* = 0.020). The Src, β-catenin and E2F3 pathways did not appear to be associated with DFS in patients with laryngeal cancer. Corresponding Kaplan-Meier curves are shown in Figure 5. Finally, we performed multivariate analysis, including the 28-gene model and the Ras pathway and found that the predictor maintained its independent prognostic significance (Wald's *p* = 0.001), as did the oncogenic pathway (Table 6).

**Table 6:** Univariate and multivariate analyses - Ras pathway.

	Univariate *p* value	Hazard Ratio (HR)	95% CI		Multivariate *p* value	Hazard Ratio (HR)	95% CI	
			Lower	Upper			Lower	Upper
**28-gene model**	<0.001	4.58	2.06	10.19	0.001	4.16	1.86	9.32
**Ras pathway**	0.020	2.55	1.91	5.45	0.036	2.26	1.06	4.85

### qPCR validation of a subset of genes from the 28-gene predictor

**Figure 6:**
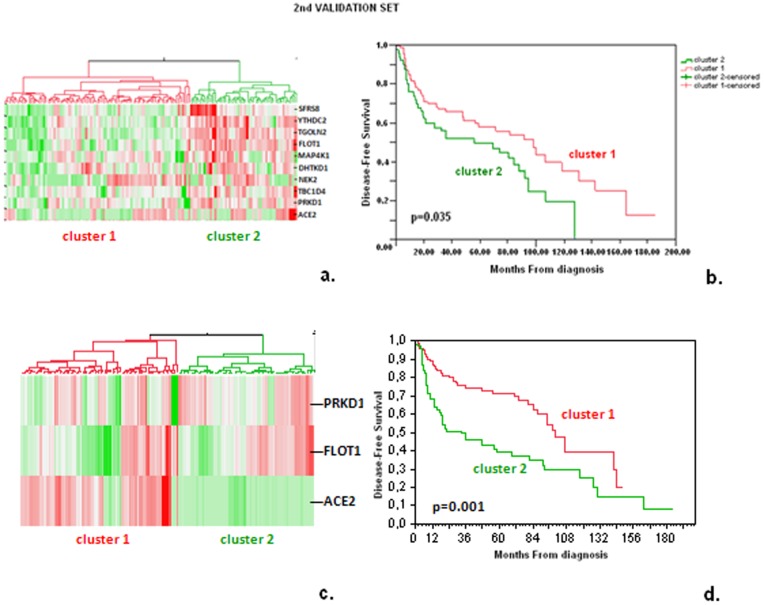
Independent validation of selected genes from the 28-gene model. Hierarchical clustering of RQ values from the genes tested in the 2^nd^ validation set (FFPE samples). In panels a and b, RQ values of the 10 applicable genes were evaluated. Two major clusters were identified (a) that were shown to have a significant effect on patient DFS (b). Cluster 1 was associated with a better prognosis compared to cluster 2. However, the majority of these genes were clustered in the opposite direction than that expected from the 28-gene classifier. In panels c and d, clustering of the 3 genes with individually significant associations yielded 2 patient groups with distinct outcome; in comparison to the original 28-gene classifier, correct patterns of gene expression were present in the corresponding good and bad prognosis groups. Red and green colors in panels a and c denote high and low expression, respectively.

The aforementioned analyses were performed using fresh-frozen tissue samples. However, this approach has certain methodological limitations when it comes to daily clinical practice. Thus, we attempted to validate our multigene predictor using qPCR on FFPE tumor tissue samples, a more easily applicable methodology. As described in the Materials and Methods section, however, it was possible to reliably investigate in our FFPE sample series the expression of only 10 of the 28 genes in the original predictor. The descriptive characteristics of RQ values obtained from the informative samples per assay are described in Table 7 (means, medians, SD, min, max). At first, we attempted to cluster continuous RQ values for all these genes. However, hierarchical clustering did not yield results comparable to the 28-gene predictor in a meaningful way, with the majority of high and low RQ values clustered in the opposite direction (Figures 6a and b). This was of no surprise, because we only examined 1/3 of the genes probed in the array signature, while fold-change in the expression of these genes was very narrow and could easily be reversed with a different method (qPCR vs. array hybridization) or a different type of material (FFPE vs. fresh-frozen tissues). Detailed analytical and statistical approaches for the behavior of each qPCR assay vs. the array probe in matched FFPE vs. fresh-frozen samples would be needed for the clarification of this discrepancy, however such an in-depth analysis was beyond the scope of the present study. Therefore, we next applied pre-determined profiling for the investigation of possible effects of the genes tested with qPCR on patient DFS. For this purpose, we transformed continuous RQ values into binary parameters (high/low expression). Based on the narrow fold-change of gene expression between the good and bad prognosis groups in the 28-gene predictor, we used median RQ values to classify high and low expression for the 10 evaluable genes. Log-rank testing for these genes as single markers yielded associations with outcome similar to those observed for the corresponding genes in the U133A signature (high/low patterns comparable with up- and down-regulation of gene expression, respectively, for 6 of the 10 genes), some of which were significant (PRKD1) or showed a trend for significance (ACE2, FLOT1) (Table 8). Hierarchical clustering of continuous RQ values for the latter three genes yielded two groups of patients with significantly different DFS (Figures 6c and d). The expected gene expression pattern was present in the correct context with outcome, but was absent in the majority of cases.

**Table 7:** Gene expression characteristics for the 10 evaluable mRNA targets investigated with qPCR in 187 FFPE samples.

	ACE2	DHTKD1	FLOT1	MAP4K1	NEK2	SFRS8	PRKD1	TBC1D4	TGOLN2	YTHDC2
**N (informative)**	159	171	178	169	156	184	162	165	183	176
**% (informative samples)**	85.03	91.44	95.19	90.37	83.42	98.4	86.63	88.24	97.86	94.12
**Mean**	38.8	46.3	47.03	45.63	39.37	47.62	44.25	44.68	46.95	46.11
**Median**	36	46.48	47.01	45.76	39.38	47.54	44.58	44.97	46.85	46.21
**Std. Deviation**	4.09	1.68	0.86	1.79	3.44	0.51	2.12	2.21	0.63	1.33
**Minimum**	32.26	36	44.81	36	33.49	46.02	36	35.27	45.4	36
**Maximum**	53.75	50.16	49.26	52.06	47.08	49.41	47.88	49.36	50.21	49.43

**Table 8:** Individual and profiled gene expression effects on DFS.

	N patients	Median (mo)	SD	95% CI, low	95% CI, high	Log-rank p	Gene expression in the good prognosis signature (30-gene predictor)	Concordance (trend) with the 30-gene predictor pattern
**ACE2**	**159**					**0.070**	Up-regulated	YES
low	71	56.52	23.23	10.99	102.06			
high	88	97.87	7.36	83.44	112.30			
**DHTKD1**	**171**					0.436	Up-regulated	NO
low	85	94.46	15.75	63.60	125.32			
high	86	83.77	11.86	60.52	107.02			
**FLOT1**	**178**					**0.068**	Down-regulated	YES
low	89	100.30	12.55	75.69	124.90			
high	89	80.46	26.01	29.48	131.44			
**MAP4K1**	**169**					0.252	Up-regulated	YES
low	86	74.11	23.75	27.55	120.67			
high	83	91.80	9.12	73.92	109.69			
**NEK2**	**156**					0.366	Down-regulated	YES
low	78	94.43	7.48	79.76	109.10			
high	78	69.15	18.68	32.53	105.76			
**PRKD1**	**162**					**0.012**	Down-regulated	YES
low	81	94.46	7.75	79.28	109.64			
high	81	69.15	27.42	15.41	122.89			
**SFRS8**	**184**					0.400	Up-regulated	NO
low	92	91.15	17.82	56.21	126.08			
high	92	87.34	11.77	64.27	110.41			
**TBC1D**	**165**					0.193	Up-regulated	NO
low	82	91.15	15.67	60.44	121.85			
high	82	74.11	13.92	46.82	101.40			
**TGOLN2**	**183**					0.360	Down-regulated	YES
low	91	91.15	15.07	61.61	120.69			
high	92	82.82	20.67	42.30	123.34			
**YTHDC2**	**176**					0.149	Up-regulated	NO
low	88	97.87	15.64	67.22	128.51			
high	88	80.46	19.87	41.51	119.41			
**ACE2/FLOT1/PRKD1**	**149**					**0.009**	N/A	
intermediate profile	108 (72.5%)	83.77	15.32	53.75	113.79			
high/low/low	20	141.84	49.20	45.41	238.26			YES, good prognosis
low/high/high	21	13.93	8.33	0.00	30.26			YES, bad prognosis
**FLOT1/PRKD1**	**161**					**0.006**	N/A	
intermediate profile	78 (48.4%)	91.15	6.51	78.38	103.91			
low/low	42	141.84	65.83	12.81	270.86			YES, good prognosis
high/high	41	46.75	30.24	0.00	106.03			YES, bad prognosis
**ACE2/PRKD1**	**149**					**0.005**	N/A	
intermediate profile	72 (48.3%)	87.34	16.82	54.38	120.31			
high/low	36	141.84	26.43	90.03	193.65			YES, good prognosis
low/high	41	18.79	4.13	10.68	26.89			YES, bad prognosis

N/A, non-applicable.

Significant p values are shown in bold.

Application of the good prognosis high/low gene expression patterns to the binary transformed RQ values revealed no single tumor with the expected pattern for all 10 genes. However, profiling of the three significant genes only revealed a strong association with patient outcome (univariate COX regression, overall Wald's *p* = 0.012). In particular, tumors expressing ACE2 high, FLOT1 and PRKD1 low (expected good prognosis profile according to the 30-gene predictor, n = 20) were indeed associated with a significantly longer DFS, as compared to tumors expressing ACE2 low, FLOT1 and PRKD1 high (expected bad prognosis profile, n = 21) (good vs. bad profile, HR = 0.24, 95% CI 0.09–0.63, Wald's *p* = 0.003). As with hierarchical clustering for the three genes, the majority of tumors (n = 108) did not fit into the above profiles and were of intermediate prognosis with a trend to perform better than the tumors expressing the bad profile (intermediate vs. bad HR = 0.58, 95% CI 0.33–1.03, Wald's *p* = 0.063). Similar results were obtained upon profiling PRKD1 with ACE2 (univariate Cox, overall Wald's p = 0.006; expected good vs. bad profile HR = 0.35, 95% CI 0.18–0.68, *p* = 0.002), and PRKD1 with FLOT1 (univariate Cox, overall Wald's p = 0.013; expected good vs. bad profile HR = 0.39, 95% CI 0.21–0.73, Wald's p = 0.003) (Figure 5, Table 8). A more detailed sub-classification of tumors according to more combinations of high/low for the above genes did not yield significant associations with outcome.

With respect to clinicopathological parameters, no association was observed for age, alcohol consumption, smoking habits, histological grade, stage, or lymph node status, with ACE2, FLOT1 and PRKD1 mRNA as single binary variables, as clusters, or as pre-determined profiles. High ACE2 expression was observed more often in tumors without lymph node involvement (66 out of 137 [48.2%]), as compared to tumors with positive lymph nodes (5 out of 22 [22.7%], Fisher's exact test *p* = 0.036). In comparison, the unfavorable prognosis cluster with continuous RQ values for the three genes was more often found in tumors with positive (14 out of 19 [73.7%]) than in tumors with negative lymph nodes (55 out of 130 [42.3%], Wald's *p* = 0.013).

Binary ACE2, FLOT1, PRKD1 variables, ACE2/FLOT1/PRKD1, ACE2/PRKD1 and FLOT1/PRKD1 profiles, as well as ACE2/FLOT1/PRKD1 clusters were also tested for their effect on patient outcome in multivariate models, along with age, alcohol consumption, smoking status, lymph node status, extent of surgery and post-operative radiation. High PRKD1 mRNA expression as a single marker (HR = 2.00, 95% CI 1.28–3.14, Wald's *p* = 0.002) and positive lymph node status (HR = 4.00, 95% CI 2.22–7.37, Wald's *p*<0.001) independently predicted for unfavorable DFS, while patients that had undergone total laryngectomy had decreased risk for relapse (HR = 0.55, 95% CI 0.31–0.95, Wald's *p* = 0.036), as compared to all other surgical approaches.

## Discussion

Our group has previously identified prognostic gene models in patients with early stage (T1N0M0, T2N0M0) laryngeal cancer [28] by using Illumina expression profiling (Illumina, CA) in 56 FFPE tissue samples. While this former study provided promising information on the genetic profile of early stage laryngeal cancer, herein we sought to expand our research to operable laryngeal cancer beyond stage. The present study employed a different platform for expression profiling (Affymetrix) and two independent validation sets. We observed that the prognostic profiles from our previous work do not share the same genes with the profiles presented here. However, the present 28-gene prognostic profile successfully stratified patients of the previously published early stage laryngeal cancer cohort with respect to disease-free survival (Figure S1). We believe therefore, that even though these profiles comprise of different genes, they capture the underlying biology of the tumors, which correlates with their aggressive behavior.

Such a prognostic signature in laryngeal cancer patients has important clinical implications. Patients with unfavorable prognosis, as identified by our multigene predictor, might gain great benefit from aggressive adjuvant treatment, while patients with favorable prognosis could be spared the side effects of what would appear to be an unnecessary treatment. Large-scale, prospective studies are needed however, to shed additional light into this matter and validate our findings.

Treatment efficacy of established therapeutic modalities could be further improved by novel therapeutic approaches. In an attempt to provide hypotheses on tumor progression, as well as novel therapeutic concepts, we explored the molecular biology of laryngeal tumors. First, using Submap, we illustrated that high- and low-risk tumors in the training set are molecularly homogeneous to their respective groups in the validation set. Then, using large-scale gene expression analysis, we demonstrated that tumors of high-risk patients are characterized by deregulation of several pathways of interest. Many of these gene networks have already been identified to be prognostic in cancer. Specifically, deregulation of the TGF-beta [30], [31], VEGF [32], [33], [34], mTOR [35], Wnt [36], Hedgehog [37], [38] and insulin [39] signaling pathways has been found to be associated with aggressive disease in squamous cell carcinomas. Based on these data, the Hellenic Cooperative Oncology Group (HeCOG) proceeded with evaluating the prognostic value of the VEGF [40], EGFR [41] and insulin [42] signaling pathways in laryngeal cancer. Prospective validation of the prognostic role of the aforementioned pathways will promote the accurate identification of patients at high risk for recurrence who may necessitate a more aggressive treatment.

A number of studies also suggest that many of the genes playing a key role in these pathways may serve as novel therapeutic targets. For instance, in vivo studies in mice, targeting the mTOR [43] and IGF [44] molecules, have shown promising results for head and neck cancer treatment. Finally, it is important to outline the presence of the “Focal Adhesion” pathway in the gene set analysis results both in the training and 1^st^ validation sets [29]. Thurlow et al have previously demonstrated the prognostic significance of this network, in patients with head and neck cancer.

To further explore networks in laryngeal cancer, we sought to examine pathway activation status in each individual patient of our study. The Ras pathway appeared to be more frequently activated in tumors from high-risk patients. More importantly, there was a significant survival difference between patients with Ras pathway activation and those without. It has already been suggested that the Ras pathway plays a growth promoting role in head and neck cancer [45]. These data indicate that members of the Ras pathway may be valuable targets for chemotherapeutic interventions in laryngeal cancer.

In the present study, the identification and independent validation of multigene predictors was performed using fresh-frozen tumors from patients with resectable laryngeal cancer. However, gene expression profiling using microarray technology has several limitations that have as yet hampered its introduction into daily clinical practice. These include the need for fresh-frozen tissue, the lack of reproducibility and external validation, the complexity of microarray data analysis and cost. In an effort to transcribe our prognostic signature into clinically applicable markers, we tested genes from this classifier on a series of routinely processed FFPE samples from laryngeal cancer patients with similar disease characteristics at presentation. However, in order to evaluate the effectiveness of this validation step, as it is presented here, we need to consider that the 2^nd^ validation set differed from the other two study groups in the type of tissue material used and the method applied for gene expression profiling. FFPE RNA quantification results may differ in comparison to those from frozen tissues [15], [46], while microarray expression signatures are usually filtered down to a few genes for qPCR applications [47]. As a result, it is challenging to reproduce the significance obtained with array classifiers. For example, Mirisola et al developed a 4-gene classifier out of 213 genes distinguishing laryngeal carcinomas with and without relapse, but managed to reproduce only one of these gene markers with qPCR [15]. Herein, we applied very strict criteria for the selection of PCR targets in order to obtain profiles that would reliably reflect the ones included in the original U133 model. It should also be noted that, in comparison to the training and 1^st^ validation cohorts, patients in the 2^nd^ validation cohort experienced a higher incidence of recurrence. With all the above restrictions and reservations it was possible to validate profiles for 3 of the 28 genes in the array predictor, i.e., ACE2, FLOT1 and PRKD1. Despite the fact that 49–74% of tumors would fall into the intermediate category of undetermined prognosis, combinations of these markers might serve for distinguishing laryngeal cancer patients who would remain disease-free for more than 10 years or who would experience early relapse. Importantly, out of these three genes, PRKD1 was revealed to be an independent predictor for outcome. Hence, testing for PRKD1 expression only might, in fact, be more efficient for predicting laryngeal cancer prognosis than testing for all three genes. These are novel findings, since none of ACE1, FLOT1 and PRKD1 have previously been investigated in laryngeal or head and neck cancer, neither is it known whether these genes are functional in the normal laryngeal epithelium. The latter findings however, should be viewed as hypothesis generating rather than definitive.

From the biological point of view, the expression patterns of ACE2, FLOT1 and PRKD1 in association with good or bad prognosis are largely in line with the proposed anti-growth and anti-tumor roles for ACE2 [48], ; with tumor promoting roles for FLOT1 [50], [51]; and, with pro-metastatic roles for PRKD1 [52], [53], [54]. It should be noted, however, that the differences observed in the expression of ACE2, FLOT1 and PRKD1 in favourable and unfavourable laryngeal carcinomas were not clear-cut in the array predictor, which may reflect the lack of direct genomic alterations within the corresponding gene sequences. Determining RQ value cut-offs for prospective use remains a challenge with all qPCR expression markers of this type. Based on the present results, it seems worthy pursuing PRKD1 expression at the protein level with immunohistochemistry for routine practice, provided that validated antibodies for this marker will become commercially available.

One potential criticism of the analyses presented here is that patients have been treated over a long period of time and that treatment modalities might have changed over these years. However, laryngeal cancer is a rare disease and it would be difficult to acquire a large number of samples from patients treated over a shorter period of time. Another limitation is that patients included in this study have been treated in a singe institution. For this reason, one might question the applicability of our multigene predictors in diverse patient populations. Large-scale multicenter studies are therefore necessary to confirm the applicability of our prognostic models.

Moreover, all of our patients were treated with surgery, while approximately 40% of the patients also received radiotherapy. Of note, current treatment guidelines in many European countries [17], in early stages of laryngeal cancer, include surgical resection with or without adjuvant radiotherapy, which renders the findings of the present study both timely and clinically relevant. It is unclear however, whether patients with an unfavorable prognostic profile would be better treated with chemoradiotherapy, as given in other countries including the US [18], or other approaches. Prospective studies are needed in order to clarify this issue.

## Conclusions

To summarize, laryngeal cancer is a heterogeneous disease. Patients with similar clinicopathological features may have a different outcome. It is important therefore, to accurately identify which patients with laryngeal cancer will recur, in order to use more aggressive and effective modalities. Our model accurately identified patients at high-risk for recurrence in the training set, as well as, in two independent validation sets. This model does not appear to solely be a collection of prognostic genes, but provides insight into disease mechanisms and potential therapeutic targets. The prognostic and biological significance of ACE2, FLOT1 and especially PRKD1 merits prospective validation, in order for these factors to possibly serve as independent prognostic markers for recurrence in patients with surgically resected squamous-cell carcinoma of the larynx.

## Supporting Information

Figure S1 **External validation of our 28-gene model using publicly available data.** Kaplan-Meier survival estimates for high- and low-risk patients, as predicted by our 28-gene model in a separate cohort of patients with early stage laryngeal cancer.
(DOC)

File S1 **Kaplan-Meier survival estimates for high- and low-risk patients, as defined by prognostic models comprising 20 to 250 probe sets.**
(DOC)

Table S1 **Detailed clinical characteristics for individual patients in the training set and the 1^st^ and 2^nd^ validation sets.**
(DOC)

Table S2 **Gene set analysis in patients with poor and good prognosis in the training and 1^st^ validation sets.**
(DOC)
