# Supplementary figures and images for: Identification and Validation of a Multigene Predictor of Recurrence in Primary Laryngeal Cancer
Source: PLoS One. 2013 Aug 9;8(8):e70429. doi: 10.1371/journal.pone.0070429 (PMC3739775; doi:10.1371/journal.pone.0070429)

**
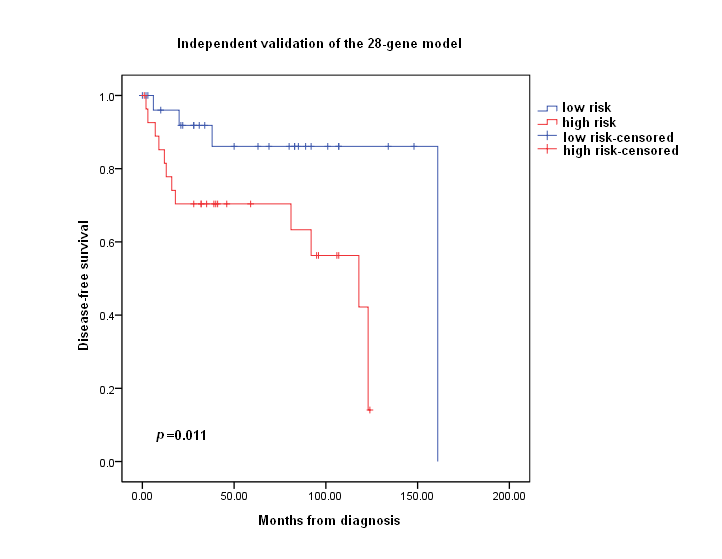
**

Supplement: Figure S1 — External validation of our 28-gene model using publicly available data. Kaplan-Meier survival estimates for high- and low-risk patients, as predicted by our 28-gene model in a separate cohort of patients with early stage laryngeal cancer. (DOC) [file pone.0070429.s001.doc]

**TRAINING SET**


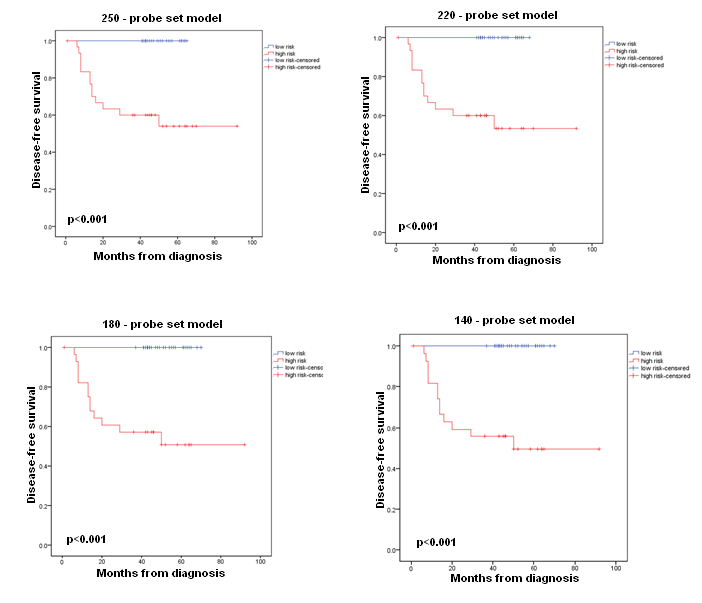


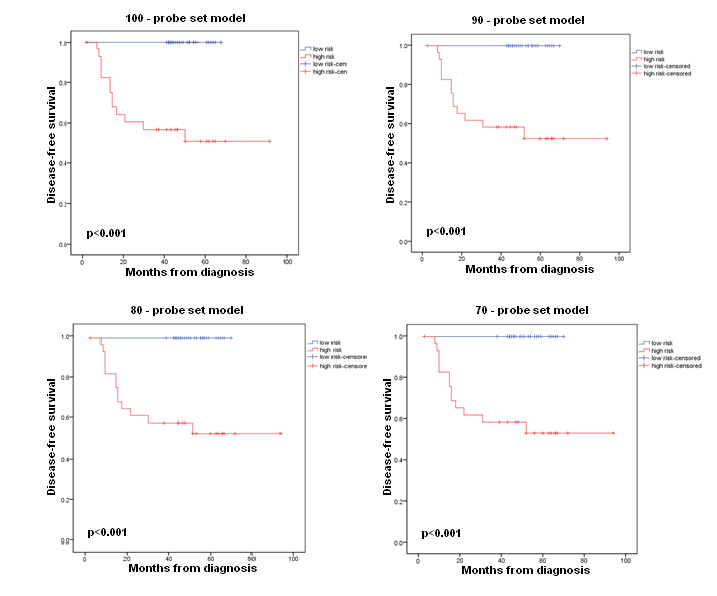


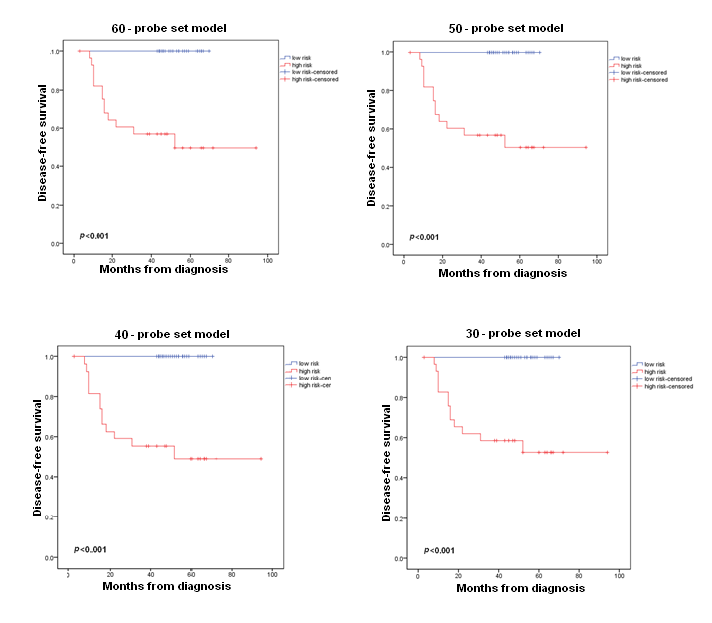


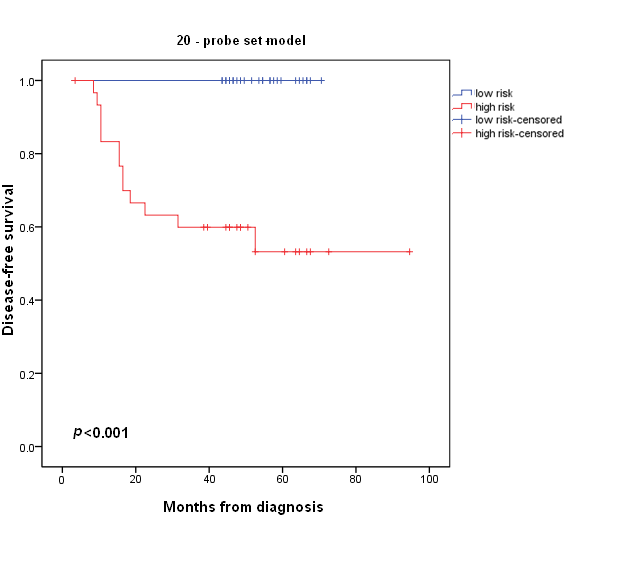


**1st VALIDATION SET**

**
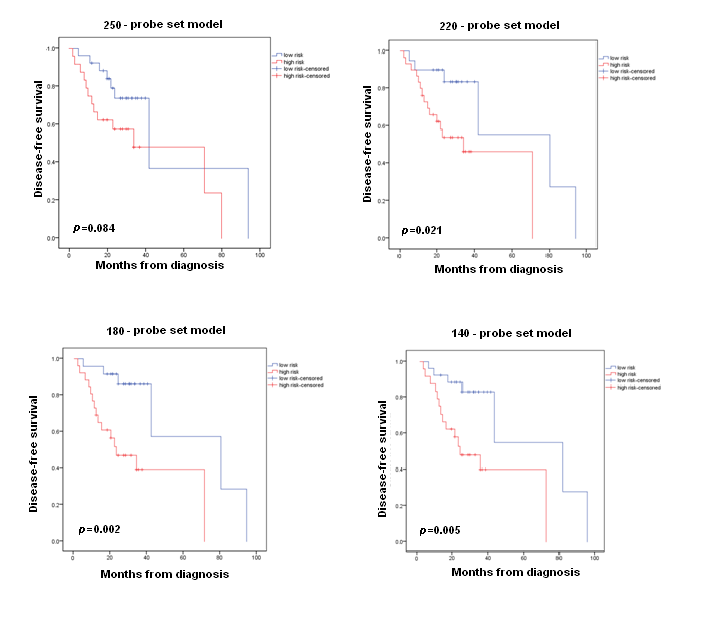
**

**
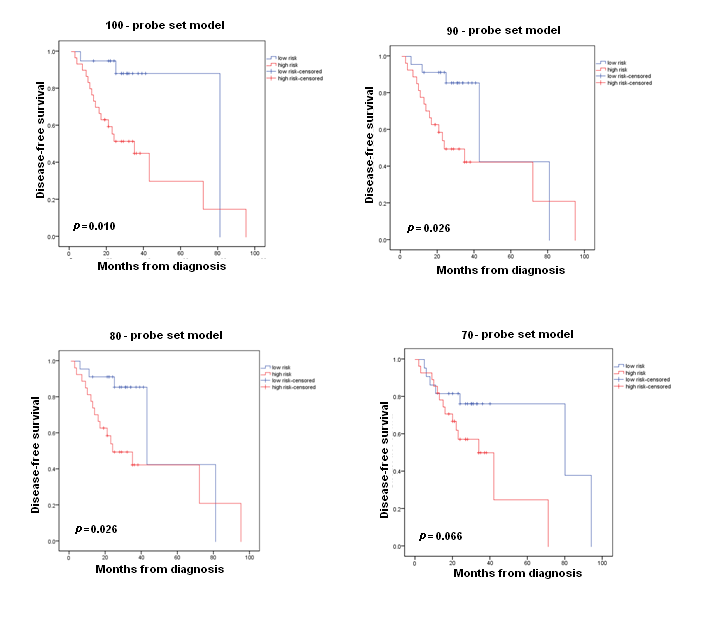
**

**
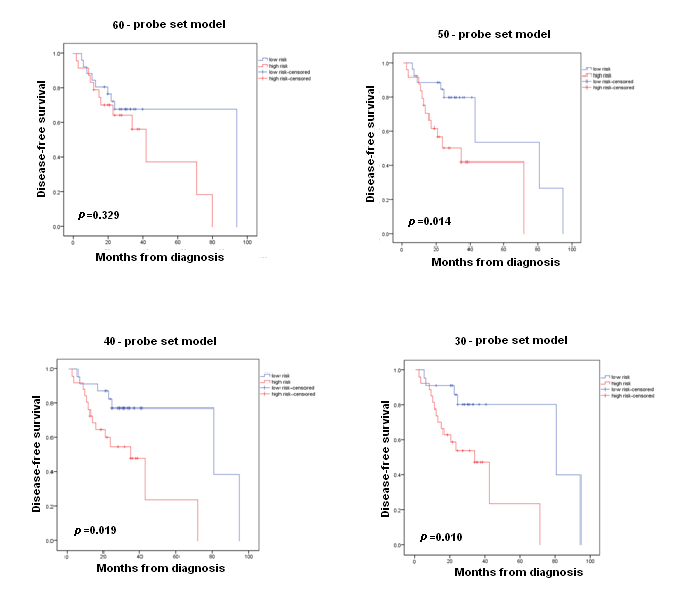
**

**
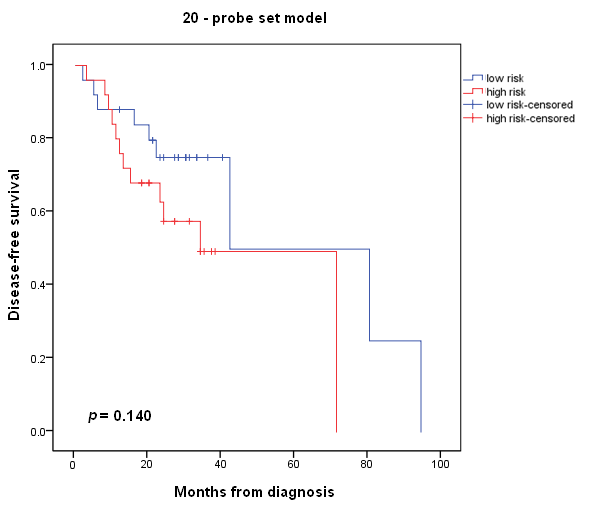
**

Supplement: File S1 — Kaplan-Meier survival estimates for high- and low-risk patients, as defined by prognostic models comprising 20 to 250 probe sets. (DOC) [file pone.0070429.s002.doc]
