# Supplementary material for: Identification and Validation of a Multigene Predictor of Recurrence in Primary Laryngeal Cancer
Source: PLoS One. 2013 Aug 9;8(8):e70429. doi: 10.1371/journal.pone.0070429 (PMC3739775; doi:10.1371/journal.pone.0070429)
Supplement: Table S1 — Detailed clinical characteristics for individual patients in the training set and the 1st and 2nd validation sets. (DOC) [file pone.0070429.s003.doc]

| **Experiment ##Names** | **Patient_no** | **Set** | **Age** | **Gender** | **Smoking** | **Alcohol** | **Stage** | **Grade** | **Recurrence** | **Radiation** | **Surgery Date** |
| --- | --- | --- | --- | --- | --- | --- | --- | --- | --- | --- | --- |
| HN2200a | A01 | training | 54 | male | yes | moderate/heavy | 3 | 1 | no | no | 08/2004 |
| HN2201a | A03 | training | 60 | male | no | no/mild | 3 | unknown | no | no | 08/2004 |
| HN2202ms | A04 | training | 69 | male | yes | moderate/heavy | 4 | 3 | yes | yes | 08/2004 |
| HN2203ms | A05 | training | 57 | male | yes | moderate/heavy | 4 | 1 | no | yes | 09/2004 |
| HN2205ms | A08 | training | 58 | male | yes | no/mild | 4 | 3 | yes | no | 09/2004 |
| HN2206ms | A11 | training | 70 | male | yes | moderate/heavy | 3 | 1 | no | no | 11/2004 |
| HN2207ms | A12 | training | 58 | male | yes | moderate/heavy | 3 | 1 | yes | yes | 11/2004 |
| HN2208ms | A13 | training | 82 | male | yes | no/mild | 4 | 2 | no | no | 12/2004 |
| HN2209ms | A14 | training | 88 | male | yes | no/mild | 3 | 2 | yes | yes | 09/2004 |
| HN2210ms | A15 | training | 64 | male | yes | moderate/heavy | 1 | 2 | no | no | 01/2005 |
| HN2211ms | A16 | training | 48 | male | yes | no/mild | 2 | 2 | no | no | 02/2005 |
| HN2213ms | A19 | training | 68 | male | yes | moderate/heavy | 2 | unknown | no | no | 02/2005 |
| HN2214ms | A20 | training | 45 | male | yes | no/mild | 4 | 2 | no | yes | 02/2005 |
| HN2215ms | A21 | training | 43 | male | yes | no/mild | 2 | 2 | no | no | 02/2005 |
| HN2216ms | A22 | training | 51 | male | yes | moderate/heavy | 2 | 1 | no | no | 03/2005 |
| HN2217ms | A23 | training | 62 | male | yes | moderate/heavy | 4 | 2 | yes | yes | 03/2005 |
| HN2218ms | A24 | training | 74 | male | yes | moderate/heavy | 2 | 2 | no |  | 04/2005 |
| HN2219ms | A25 | training | 72 | male | yes | moderate/heavy | 3 | 1 | no | no | 04/2005 |
| HN2220ms | A26 | training | 50 | male | yes | moderate/heavy | 4 | 1 | no |  | 03/2005 |
| HN2221ms | A27 | training | 48 | male | yes | no/mild | 4 | 2 | no | yes | 04/2005 |
| HN2222ms | A28 | training | 49 | male | yes | moderate/heavy | 4 | 1 | no | no | 04/2005 |
| HN2223ms | A30 | training | 65 | male | yes | no/mild | 4 | 2 | yes | yes | 06/2005 |
| HN2224ms | A31 | training | 58 | male | yes | moderate/heavy | 4 | 2 | no | yes | 07/2005 |
| HN2225ms | A32 | training | 46 | male | yes | no/mild | 3 | 1 | yes | yes | 07/2005 |
| HN2226ms | A35 | training | 70 | male | yes | moderate/heavy | 3 | 3 | no | yes | 08/2005 |
| HN2227ms | A37 | training | 72 | male | yes | moderate/heavy | 4 | 3 | no |  | 08/2005 |
| HN2228ms | A38 | training | 55 | male | yes | moderate/heavy | 3 | 2 | yes | no | 08/2005 |
| HN2229ms | A39 | training | 69 | male | yes | no/mild | 4 | 3 | no | yes | 07/2005 |
| HN2230ms | A40 | training | 55 | male | yes | no/mild | 4 | 3 | no |  | 09/2007 |
| HN2231ms | A41 | training | 50 | male | yes | no/mild | 4 | 2 | yes | yes | 09/2007 |
| HN2232ms | A42 | training | 74 | male | yes | no/mild | 4 | 3 | no | yes | 09/2007 |
| HN2233ms | A43 | training | 60 | male | yes | no/mild | 4 | 2 | no | yes | 10/2005 |
| HN2234ms | A44 | training | 71 | male | yes | moderate/heavy | 4 | 2 | no |  | 10/2005 |
| HN2235ms | A45 | training | 74 | male | yes | no/mild | 3 | 1 | no | no | 11/2005 |
| HN2236ms | A47 | training | 61 | male | yes | moderate/heavy | 4 | 3 | yes | yes | 11/2005 |
| HN2237_2ms | A49 | training | 59 | male | yes | moderate/heavy | 3 | 1 | no | yes | 11/2005 |
| HN2238_2ms | A51 | training | 68 | male | yes | no/mild | 3 | 1 | no | yes | 12/2005 |
| HN2239ms | A53 | training | 67 | male | yes | moderate/heavy | 3 | 2 | no | no | 01/2006 |
| HN2240_2ms | A54 | training | 60 | male | yes | moderate/heavy | 3 | 1 | yes | no | 01/2006 |
| HN2241ms | A55 | training | 60 | male | yes | moderate/heavy | 4 | 2 | no | yes | 10/2002 |
| HN2242ms | A56 | training | 69 | female | yes | no/mild | 4 | 2 | no | no | 02/2006 |
| HN2244ms | A58 | training | 61 | male | yes | moderate/heavy | 2 | 2 | no | no | 02/2006 |
| HN2246ms | A62 | training | 79 | male | yes | no/mild | 3 | 1 | no |  | 03/2006 |
| HN2247ms | A63 | training | 68 | male | yes | moderate/heavy | 4 | 2 | no | yes | 04/2006 |
| HN2248ms | A64 | training | 64 | male | yes | moderate/heavy | 3 | 2 | yes |  | 05/2006 |
| HN2249ms | A65 | training | 65 | male | yes | moderate/heavy | 3 | 3 | no | yes | 05/2006 |
| HN2250ms | A66 | training | 49 | male | yes | moderate/heavy | 3 | 1 | no |  | 06/2006 |
| HN2252ms | A69 | training | 67 | female | yes | no/mild | 3 | 2 | no | no | 07/2006 |
| HN2253ms | A70 | training | 66 | male | yes | moderate/heavy | 3 | 1 | no | no | 07/2006 |
| HN2254ms | A71 | training | 65 | female | yes | no/mild | 4 | 2 | no | yes | 09/2006 |
| HN2255ms | A72 | training | 80 | male | yes | moderate/heavy | 4 | 2 | no |  | 08/2006 |
| HN2257_2ms | A75 | training | 55 | male | yes | moderate/heavy | 2 | 2 | no | no | 10/2006 |
| HN2258ms | A76 | training | 49 | male | yes | moderate/heavy | 4 | 1 | no |  | 10/2006 |
| HN2259ms | A78 | training | 54 | male | yes | moderate/heavy | 2 | 1 | no | no | 10/2006 |
| HN2260ms | A79 | training | 62 | male | yes | no/mild | 3 | 2 | no | yes | 11/2006 |
| HN2261ms | A80 | training | 41 | female | yes | no/mild | 4 | 1 | no | yes | 09/2006 |
| HN2262ms | A81 | training | 68 | male | yes | no/mild | 2 | 2 | yes |  | 12/2006 |
| HN2263ms | A82 | training | 81 | male | yes | moderate/heavy | 3 | 3 | no | no | 11/2006 |
| HN2264ms | A83 | training | 67 | male | yes | no/mild | 2 | 2 | no | yes | 12/2006 |
| HN2265ms | A73 | 1st validation | 54 | male | yes | no/mild | 1 | 1 | no | no | 10/2006 |
| HN2266ms | A84 | 1st validation | 81 | male | yes | moderate/heavy | 4 | 1 | no | no | 01/2007 |
| HN2267ms | A85 | 1st validation | 67 | male | yes | no/mild | 3 | 1 | no | no | 01/2007 |
| HN2268ms | A86 | 1st validation | 72 | male | yes | no/mild | 3 | 3 | yes | yes | 01/2007 |
| HN2270ms | A88 | 1st validation | 67 | male | yes | moderate/heavy | 4 | 2 | yes | yes | 02/2007 |
| HN2271ms | A89 | 1st validation | 66 | male | yes | no/mild | 1 | 2 | no | no | 02/2007 |
| HN2272ms | A90 | 1st validation | 72 | male | yes | moderate/heavy | 4 | 2 | yes | yes | 03/2007 |
| HN2273ms | A91 | 1st validation | 64 | male | yes | moderate/heavy | 4 | 2 | no | yes | 03/2007 |
| HN2274ms | A92 | 1st validation | 69 | male | yes | no/mild | 3 | 1 | no | yes | 04/2007 |
| HN2275ms | A93 | 1st validation | 63 | male | yes | moderate/heavy | 3 | 2 | no | yes | 04/2007 |
| HN2276ms | A94 | 1st validation | 53 | male | yes | no/mild | 1 | 1 | no | no | 05/2007 |
| HN2278ms | Y35 | 1st validation | 59 | male | yes | moderate/heavy | 1 | 2 | yes | no | 11/2006 |
| HN2279ms | A96 | 1st validation | 60 | male | yes | moderate/heavy | 4 | 2 | no | yes | 05/2007 |
| HN2280ms | A97 | 1st validation | 79 | male | yes | moderate/heavy | 4 | 1 | yes | yes | 06/2007 |
| HN2281ms | A98 | 1st validation | 67 | male | yes | no/mild | 3 | 1 | no | yes | 07/2007 |
| HN2282ms | A99 | 1st validation | 67 | male | yes | moderate/heavy | 4 | 2 | no | yes | 07/2007 |
| HN2283ms | A100 | 1st validation | 56 | male | yes | moderate/heavy | 3 | 1 | no | no | 07/2007 |
| HN2284ms | A101 | 1st validation | 63 | male | yes | no/mild | 1 | 3 | no | no | 07/2007 |
| HN2285ms | A102 | 1st validation | 53 | male | yes | no/mild | 2 | 1 | no | no | 08/2007 |
| HN2286ms | A103 | 1st validation | 70 | male | yes | no/mild | 3 | 1 | no | yes | 08/2007 |
| HN2287ms | A104 | 1st validation | 48 | female | yes | no/mild | 3 | 1 | no | no | 08/2007 |
| HN2289ms | A106 | 1st validation | 60 | male | yes | moderate/heavy | 4 | 2 | yes | yes | 10/2007 |
| HN2290ms | A108 | 1st validation | 69 | male | yes | moderate/heavy | 4 | 1 | yes | yes | 10/2007 |
| HN2291ms | A109 | 1st validation | 50 | male | yes | no/mild | 2 | 1 | no | no | 10/2007 |
| HN2293ms | A111 | 1st validation | 64 | male | yes | no/mild | 2 | 2 | no | no | 11/2007 |
| HN2295ms | A113 | 1st validation | 77 | male | yes | moderate/heavy | 3 | 1 | yes | yes | 10/2007 |
| HN2296ms | A114 | 1st validation | 76 | male | yes | no/mild | 4 | 2 | no | yes | 10/2007 |
| HN2297ms | A115 | 1st validation | 60 | male | yes | moderate/heavy | 4 | 3 | no | yes | 02/2008 |
| HN2298ms | A116 | 1st validation | 73 | male | yes | no/mild | 2 | 2 | no | no | 02/2008 |
| HN2299ms | A117 | 1st validation | 56 | male | yes | moderate/heavy | 3 | 2 | no | yes | 02/2008 |
| HN2300ms | A118 | 1st validation | 63 | male | yes | moderate/heavy | 4 | 2 | no | yes | 02/2008 |
| HN2301ms | A119 | 1st validation | 78 | male | yes | moderate/heavy | 4 | 2 | no | yes | 02/2008 |
| HN2302ms | A120 | 1st validation | 57 | male | yes | moderate/heavy | 4 | 2 | no | yes | 03/2008 |
| HN2303ms | A121 | 1st validation | 72 | male | yes | no/mild | 2 | 3 | no | no | 03/2008 |
| HN2305ms | A123 | 1st validation | 69 | male | yes | moderate/heavy | 3 | 2 | yes | no | 04/2008 |
| HN2306ms | A124 | 1st validation | 82 | male | yes | no/mild | 3 | 1 | no | no | 05/2008 |
| HN2307ms | A125 | 1st validation | 60 | male | yes | no/mild | 4 | 2 | no | yes | 05/2008 |
| HN2308ms | A126 | 1st validation | 47 | male | yes | no/mild | 4 | 3 | yes | yes | 05/2008 |
| HN2310ms | A128 | 1st validation | 55 | male | yes | moderate/heavy | 3 | 1 | yes | no | 06/2008 |
| HN2312ms | A130 | 1st validation | 61 | male | yes | moderate/heavy | 4 | 3 | no | yes | 06/2008 |
| HN2313ms | A131 | 1st validation | 63 | male | yes | no/mild | 1 | 1 | yes |  | 02/2008 |
| HN2319ms | y26 | 1st validation | 62 | male | yes | moderate/heavy | 1 | 1 | yes | yes | 12/1999 |
| HN2321ms | y30 | 1st validation | 74 | male | yes | no/mild | 1 | 1 | yes | yes | 01/2005 |
| HN2322ms | y31 | 1st validation | 61 | male | yes | no/mild | 1 | 2 | yes | no | 01/2006 |
| HN2324ms | y33 | 1st validation | 70 | male | yes | moderate/heavy | 2 | 1 | yes | yes | 01/2003 |
| HN2325ms | y34 | 1st validation | 53 | male | yes | no/mild | 1 | 1 | yes | yes | 03/2005 |
| HN2326ms | y36 | 1st validation | 82 | male | yes | no/mild | 3 | 2 | yes | yes | 03/2007 |
| HN2327ms | y37 | 1st validation | 68 | male | yes | no/mild | 1 | 1 | yes | no | 05/2006 |
| HN2328ms | y38 | 1st validation | 74 | male | yes | moderate/heavy | 2 | 1 | yes | yes | 10/2001 |
| HN2329ms | y39 | 1st validation | 41 | male | yes | no/mild | 2 | 2 | yes | no | 09/1999 |
| LO002a | LO002a | 2nd validation | 47 | male | yes | moderate/heavy | 3 | 2 | yes | no | 11/1997 |
| LO003a | LO003a | 2nd validation | 58 | male | yes | no/mild | 3 | 1 | yes | no | 09/1997 |
| LO007 | LO007 | 2nd validation | 44 | male | yes | no/mild | 3 | unknown | no | no | 03/1997 |
| LO008 | LO008 | 2nd validation | 61 | male | no | moderate/heavy | 1 | 2 | yes | no | 06/1992 |
| LO010 | LO010 | 2nd validation | 59 | male | yes | moderate/heavy | 4 | 1 | yes | no | 12/1996 |
| LO011 | LO011 | 2nd validation | 63 | male | yes | no/mild | 4 | 2 | yes | yes | 12/1996 |
| LO013 | LO013 | 2nd validation | 69 | male | yes | moderate/heavy | 3 | 2 | yes | yes | 11/1996 |
| LO015 | LO015 | 2nd validation | 66 | male | yes | no/mild | 2 | 1 | yes | no | 10/1996 |
| LO016 | LO016 | 2nd validation | 57 | male | yes | no/mild | 4 | 3 | yes | yes | 10/1996 |
| LO021a | LO021a | 2nd validation | 66 | male | yes | moderate/heavy | 3 | 2 | yes | no | 07/1996 |
| 034-LO | 034-LO | 2nd validation | 46 | male | yes | moderate/heavy | 2 | 1 | no | no | 10/1994 |
| 036-LO | 036-LO | 2nd validation | 72 | male | yes | no/mild | 4 | 2 | yes | no | 06/1994 |
| 037-LOa | 037-LOa | 2nd validation | 58 | male | yes | moderate/heavy | 4 | unknown | yes | yes | 06/1994 |
| 039-LO | 039-LO | 2nd validation | 65 | male | yes | no/mild | 2 | 1 | yes | no | 05/1994 |
| 041-LOa | 041-LOa | 2nd validation | 65 | male | yes | no/mild | 4 | 1 | yes | no | 08/1992 |
| 044-LO | 044-LO | 2nd validation | 76 | male | yes | moderate/heavy | 3 | 1 | yes | no | 01/1994 |
| 046-LO | 046-LO | 2nd validation | 53 | male | yes | moderate/heavy | 4 | 2 | yes | no | 10/1993 |
| 047-LOa | 047-LOa | 2nd validation | 63 | male | yes | moderate/heavy | 3 | 1 | no | no | 09/1993 |
| LO050a | LO050a | 2nd validation | 50 | male | yes | moderate/heavy | 3 | unknown | yes | no | 07/1993 |
| 052-LOa | 052-LOa | 2nd validation | 61 | male | yes | no/mild | 4 | 1 | yes | no | 05/1993 |
| 053-LOa | 053-LOa | 2nd validation | 53 | male | yes | no/mild | 3 | 2 | no | no | 05/1993 |
| 054-LO | 054-LO | 2nd validation | 72 | male | yes | no/mild | 3 | 2 | yes | yes | 04/1993 |
| 056-LOa | 056-LOa | 2nd validation | 66 | male | yes | no/mild | 3 | 3 | yes | no | 03/1993 |
| 061-LOa | 061-LOa | 2nd validation | 45 | male | yes | no/mild | 4 | 2 | yes | yes | 01/1993 |
| 063-LOa | 063-LOa | 2nd validation | 47 | male | yes | moderate/heavy | 1 | 1 | yes | no | 08/1992 |
| 064-LOa | 064-LOa | 2nd validation | 46 | male | yes | moderate/heavy | 3 | 2 | no | no | 01/1993 |
| 065-LOa | 065-LOa | 2nd validation | 42 | male | yes | no/mild | 3 | 2 | yes | no | 01/1993 |
| LO070a | LO070a | 2nd validation | 62 | male | yes | moderate/heavy | 3 | 2 | yes | yes | 11/1999 |
| 073-LO | 073-LO | 2nd validation | 56 | male | yes | moderate/heavy | 3 | 2 | no | no | 09/1999 |
| LO074a | LO074a | 2nd validation | 65 | male | yes | no/mild | 4 | unknown | yes | yes | 06/1999 |
| LO076a | LO076a | 2nd validation | 66 | male | yes | moderate/heavy | 4 | 1 | yes | no | 05/1999 |
| LO078 | LO078 | 2nd validation | 66 | male | no | no/mild | 3 | 3 | no | no | 05/1999 |
| LO079 | LO079 | 2nd validation | 69 | male | yes | no/mild | 2 | 2 | no | no | 04/1999 |
| LO086a | LO086a | 2nd validation | 48 | male | yes | no/mild | 3 | 1 | yes | no | 09/1998 |
| LO089a | LO089a | 2nd validation | 62 | male | yes | moderate/heavy | 3 | 1 | no | no | 05/1998 |
| LO090a | LO090a | 2nd validation | 69 | male | yes | no/mild | 3 | 1 | yes | no | 04/1998 |
| LO091a | LO091a | 2nd validation | 49 | male | yes | no/mild | 2 | 1 | no | no | 03/1998 |
| LO093a | LO093a | 2nd validation | 65 | male | yes | no/mild | 3 | 2 | yes | no | 02/1998 |
| LO094a | LO094a | 2nd validation | 69 | male | yes | moderate/heavy | 4 | 3 | no | no | 01/1998 |
| LO096a | LO096a | 2nd validation | 78 | male | yes | no/mild | 3 | unknown | yes | no | 11/1997 |
| LO102a | LO102a | 2nd validation | 73 | male | no | no/mild | 4 | 3 | yes | yes | 05/2005 |
| LO112a | LO112a | 2nd validation | 69 | male | yes | moderate/heavy | 3 | 1 | no | no | 08/2004 |
| LO114 | LO114 | 2nd validation | 64 | male | yes | moderate/heavy | 4 | 3 | yes | yes | 08/2004 |
| LO115a | LO115a | 2nd validation | 49 | male | yes | no/mild | 4 | 2 | yes | yes | 07/2004 |
| LO116 | LO116 | 2nd validation | 70 | male | yes | moderate/heavy | 4 | 1 | no | no | 07/2004 |
| LO117a | LO117a | 2nd validation | 58 | male | yes | no/mild | 3 | 1 | no | no | 07/2004 |
| LO118a | LO118a | 2nd validation | 74 | male | yes | no/mild | 4 | 1 | no | no | 04/2004 |
| LO119a | LO119a | 2nd validation | 58 | male | yes | moderate/heavy | 3 | 1 | no | no | 03/2004 |
| LO121a | LO121a | 2nd validation | 47 | male | yes | no/mild | 3 | 3 | no | no | 03/2004 |
| LO123a | LO123a | 2nd validation | 57 | male | yes | moderate/heavy | 1 | 2 | yes | no | 03/1995 |
| LO125a | LO125a | 2nd validation | 76 | male | yes | no/mild | 4 | 1 | yes | yes | 12/2003 |
| LO127a | LO127a | 2nd validation | 73 | male | yes | moderate/heavy | 2 | 3 | yes | yes | 09/2002 |
| LO128 | LO128 | 2nd validation | 68 | male | yes | no/mild | 2 | 1 | yes | no | 01/1997 |
| LO133a | LO133a | 2nd validation | 54 | male | yes | moderate/heavy | 3 | unknown | no | yes | 07/2001 |
| LO136a | LO136a | 2nd validation | 57 | female | yes | no/mild | 3 | 2 | no | yes | 12/2001 |
| LO137a | LO137a | 2nd validation | 57 | male | yes | no/mild | 4 | 2 | no | no | 02/2002 |
| LO138 | LO138 | 2nd validation | 58 | male | yes | no/mild | 4 | 2 | yes | yes | 06/1988 |
| LO139a | LO139a | 2nd validation | 65 | male | yes | moderate/heavy | 1 | 2 | yes | no | 11/2000 |
| LO140a | LO140a | 2nd validation | 75 | male | yes | moderate/heavy | 4 | 2 | no | yes | 04/2002 |
| LO141a | LO141a | 2nd validation | 63 | male | yes | moderate/heavy | 4 | 3 | no | no | 04/2002 |
| LO142a | LO142a | 2nd validation | 74 | male | yes | moderate/heavy | 4 | 3 | yes | no | 04/2002 |
| LO143a | LO143a | 2nd validation | 58 | male | yes | moderate/heavy | 3 | 2 | yes | no | 04/2002 |
| LO144a | LO144a | 2nd validation | 63 | male | yes | moderate/heavy | 3 | 2 | yes | no | 06/2002 |
| LO145a | LO145a | 2nd validation | 71 | male | yes | no/mild | 4 | 2 | no | no | 06/2002 |
| LO147a | LO147a | 2nd validation | 64 | male | yes | no/mild | 3 | 2 | no | yes | 08/2002 |
| LO149a | LO149a | 2nd validation | 68 | male | yes | moderate/heavy | 3 | 2 | no | no | 09/2002 |
| LO150a | LO150a | 2nd validation | 65 | male | yes | no/mild | 4 | 2 | yes | no | 10/2002 |
| LO152 | LO152 | 2nd validation | 72 | male | yes | moderate/heavy | 3 | 2 | yes | no | 01/2001 |
| LO159a | LO159a | 2nd validation | 75 | male | yes | no/mild | 3 | 2 | yes | no | 05/2001 |
| LO160a | LO160a | 2nd validation | 70 | male | yes | moderate/heavy | 3 | 2 | no | no | 05/2001 |
| LO165a | LO165a | 2nd validation | 48 | male | yes | no/mild | 3 | 2 | no | no | 03/2000 |
| LO166a | LO166a | 2nd validation | 65 | male | yes | moderate/heavy | 4 | 1 | yes | no | 03/2000 |
| LO167a | LO167a | 2nd validation | 62 | male | yes | moderate/heavy | 3 | 2 | no | yes | 05/2000 |
| LO173a | LO173a | 2nd validation | 58 | male | yes | no/mild | 3 | 1 | no | no | 11/2000 |
| LO174 | LO174 | 2nd validation | 60 | male | yes | no/mild | 3 | 1 | no | yes | 08/2000 |
| LO178a | LO178a | 2nd validation | 56 | male | yes | moderate/heavy | 1 | 3 | yes | no | 05/2002 |
| LO181 | LO181 | 2nd validation | 55 | male | yes | moderate/heavy | 3 | 1 | yes | no | 08/1994 |
| LO183 | LO183 | 2nd validation | 66 | male | yes | moderate/heavy | 3 | 3 | no | yes | 08/2000 |
| LO185 | LO185 | 2nd validation | 71 | male | yes | moderate/heavy | 4 | 2 | no | no | 08/2003 |
| LO190 | LO190 | 2nd validation | 67 | male | yes | no/mild | 3 | 2 | no | no | 04/1999 |
| LO192a | LO192a | 2nd validation | 65 | male | yes | no/mild | 1 | 3 | yes | no | 07/2000 |
| LO194a | LO194a | 2nd validation | 58 | male | yes | no/mild | 4 | 3 | yes | no | 01/1995 |
| LO199 | LO199 | 2nd validation | 54 | male | yes | no/mild | 4 | 1 | yes | yes | 05/1995 |
| LO201a | LO201a | 2nd validation | 68 | male | yes | no/mild | 2 | unknown | no | no | 07/1996 |
| LO228 | LO228 | 2nd validation | 58 | male | yes | no/mild | 2 | 1 | yes | no | 12/2000 |
| LO230 | LO230 | 2nd validation | 71 | male | yes | no/mild | 4 | 3 | no | no | 09/2001 |
| LO232 | LO232 | 2nd validation | 70 | male | yes | no/mild | 3 | 1 | no | no | 04/2001 |
| LO234 | LO234 | 2nd validation | 76 | male | yes | no/mild | 4 | 1 | yes | no | 10/2000 |
| LO235 | LO235 | 2nd validation | 54 | male | yes | no/mild | 4 | 3 | yes | yes | 11/2000 |
| LO237 | LO237 | 2nd validation | 58 | male | yes | no/mild | 2 | 2 | yes | no | 04/1999 |
| LO241 | LO241 | 2nd validation | 53 | male | yes | no/mild | 2 | 2 | no | no | 10/2000 |
| LO242 | LO242 | 2nd validation | 45 | female | yes | no/mild | 3 | 3 | yes | yes | 09/2000 |
| LO245 | LO245 | 2nd validation | 53 | male | yes | no/mild | 1 | 2 | yes | no | 03/1995 |
| LO246 | LO246 | 2nd validation | 53 | male | yes | moderate/heavy | 4 | 1 | yes | yes | 10/2000 |
| LO248 | LO248 | 2nd validation | 72 | male | yes | moderate/heavy | 1 | 1 | yes | no | 01/1999 |
| LO249 | LO249 | 2nd validation | 51 | male | yes | moderate/heavy | 3 | 3 | no | no | 03/2002 |
| LO253 | LO253 | 2nd validation | 61 | male | yes | moderate/heavy | 3 | 2 | no | no | 10/2000 |
| LO256 | LO256 | 2nd validation | 59 | male | yes | moderate/heavy | 4 | 1 | yes | no | 05/2002 |
| LO268 | LO268 | 2nd validation | 64 | male | yes | no/mild | 3 | 2 | yes | no | 06/2002 |
| LO269 | LO269 | 2nd validation | 63 | male | yes | no/mild | 3 | 3 | no | no | 06/1997 |
| LO271 | LO271 | 2nd validation | 67 | female | no | no/mild | 4 | 1 | no | yes | 01/2002 |
| LO275 | LO275 | 2nd validation | 66 | male | yes | moderate/heavy | unknown | 1 | no | no | 07/2006 |
| LO287 | LO287 | 2nd validation | 72 | male | yes | no/mild | 4 | 2 | no | yes | 01/2007 |
| LO296 | LO296 | 2nd validation | 72 | male | yes | moderate/heavy | unknown | 2 | yes | yes | 03/2007 |
| LO299 | LO299 | 2nd validation | 67 | male | yes | no/mild | 3 | 1 | no | yes | 12/2006 |
| LO315a | LO315a | 2nd validation | 80 | male | yes | moderate/heavy | unknown | 2 | yes | yes | 04/2008 |
| LO318 | LO318 | 2nd validation | 60 | male | yes | moderate/heavy | unknown | 2 | yes | yes | 10/2007 |
| LO320a | LO320a | 2nd validation | 50 | male | yes | no/mild | unknown | 1 | no | no | 10/2007 |
| LO001a | LO001a | 2nd validation | 66 | male | yes | no/mild | 3 | 3 | no | yes | 11/1997 |
| LO004 | LO004 | 2nd validation | 58 | male | yes | no/mild | 3 | 2 | no | no | 09/1997 |
| 033-LOa | 033-LOa | 2nd validation | 52 | male | yes | moderate/heavy | 3 | 1 | yes | yes | 03/1995 |
| 038-LO | 038-LO | 2nd validation | 58 | male | yes | no/mild | 2 | 1 | no | no | 05/1994 |
| 042-LO | 042-LO | 2nd validation | 70 | male | yes | no/mild | 4 | 2 | yes | no | 02/1994 |
| 072-LOa | 072-LOa | 2nd validation | 50 | male | yes | moderate/heavy | 4 | 2 | yes | yes | 09/1999 |
| LO075a | LO075a | 2nd validation | 56 | male | yes | moderate/heavy | 2 | unknown | no | no | 06/1999 |
| LO080a | LO080a | 2nd validation | 78 | male | no | moderate/heavy | 3 | 1 | yes | no | 03/1999 |
| LO085a | LO085a | 2nd validation | 66 | male | yes | no/mild | 3 | 2 | no | yes | 10/1998 |
| LO107 | LO107 | 2nd validation | 62 | male | yes | no/mild | 2 | 3 | no | no | 03/2005 |
| LO129 | LO129 | 2nd validation | 50 | female | yes | no/mild | 3 | 2 | no | no | 11/2003 |
| LO134a | LO134a | 2nd validation | 76 | male | yes | moderate/heavy | 3 | 1 | yes | no | 11/2001 |
| LO177 | LO177 | 2nd validation | 62 | male | yes | moderate/heavy | 3 | 1 | no | no | 10/2002 |
| LO186a | LO186a | 2nd validation | 44 | male | yes | moderate/heavy | 3 | 1 | no | no | 01/1999 |
| LO187a | LO187a | 2nd validation | 46 | male | yes | moderate/heavy | 4 | 2 | no | no | 03/1999 |
| LO188a | LO188a | 2nd validation | 65 | male | yes | moderate/heavy | 2 | unknown | no | no | 01/1999 |
| LO262 | LO262 | 2nd validation | 61 | male | yes | no/mild | 2 | 2 | no | no | 11/2000 |
| LO273 | LO273 | 2nd validation | 50 | male | yes | moderate/heavy | 3 | 2 | yes | no | 07/2006 |
| LO282 | LO282 | 2nd validation | 59 | male | yes | no/mild | 3 | 2 | no | yes | 12/2006 |
| LO317 | LO317 | 2nd validation | 70 | male | yes | no/mild | unknown | 1 | no | yes | 08/2007 |
| LO005 | LO005 | 2nd validation | 59 | female | no | no/mild | 3 | unknown | yes | no | 08/1997 |
| LO009 | LO009 | 2nd validation | 58 | male | yes | no/mild | 4 | 2 | yes | yes | 01/1997 |
| LO012a | LO012a | 2nd validation | 60 | male | yes | no/mild | 3 | 1 | no | no | 12/1996 |
| LO025a | LO025a | 2nd validation | 56 | male | yes | moderate/heavy | 4 | 3 | yes | yes | 01/1996 |
| LO026a | LO026a | 2nd validation | 69 | male | yes | no/mild | 4 | 3 | yes | no | 12/1995 |
| LO081 | LO081 | 2nd validation | 60 | male | yes | no/mild | 2 | 1 | no | no | 11/1998 |
| LO084 | LO084 | 2nd validation | 64 | male | yes | moderate/heavy | 3 | 2 | yes | no | 10/1998 |
| LO087 | LO087 | 2nd validation | 69 | male | yes | moderate/heavy | 4 | 2 | yes | no | 08/1998 |
| LO095a | LO095a | 2nd validation | 44 | male | yes | no/mild | 2 | 1 | no | no | 12/1997 |
| LO120a | LO120a | 2nd validation | 69 | male | no | no/mild | 4 | 1 | yes | yes | 03/2004 |
| LO122a | LO122a | 2nd validation | 75 | male | yes | no/mild | 3 | 2 | yes | no | 02/2004 |
| LO154a | LO154a | 2nd validation | 55 | male | yes | moderate/heavy | 3 | 2 | yes | no | 03/2001 |
| LO155a | LO155a | 2nd validation | 55 | male | yes | no/mild | 4 | 3 | yes | yes | 03/2001 |
| LO156a | LO156a | 2nd validation | 60 | male | yes | moderate/heavy | 4 | 2 | yes | yes | 04/2001 |
| LO158a | LO158a | 2nd validation | 47 | male | yes | no/mild | 3 | 1 | no | no | 05/2001 |
| LO161a | LO161a | 2nd validation | 71 | male | yes | no/mild | 4 | 1 | no | yes | 05/2001 |
| LO191 | LO191 | 2nd validation | 72 | male | no | moderate/heavy | 4 | 2 | yes | no | 03/2003 |
| LO198a | LO198a | 2nd validation | 50 | male | yes | moderate/heavy | 1 | 3 | yes | yes | 10/1994 |
| LO244 | LO244 | 2nd validation | 73 | male | yes | no/mild | 4 | 1 | no | no | 03/2002 |
| LO252 | LO252 | 2nd validation | 62 | male | yes | moderate/heavy | 3 | 1 | yes | no | 04/2000 |
| LO254 | LO254 | 2nd validation | 72 | male | yes | moderate/heavy | 2 | 1 | yes | no | 12/1999 |
