# Supplementary material for: Identification and Validation of a Multigene Predictor of Recurrence in Primary Laryngeal Cancer
Source: PLoS One. 2013 Aug 9;8(8):e70429. doi: 10.1371/journal.pone.0070429 (PMC3739775; doi:10.1371/journal.pone.0070429)
Supplement: Table S2 — Gene set analysis in patients with poor and good prognosis in the training and 1st validation sets. (DOC) [file pone.0070429.s004.doc]

| **GENE SET ANALYSIS (GSA)** | **TRAINING SET** | **1^st^ VALIDATION SET** |
| --- | --- | --- |
| **Pathways - Gene Networks** | **Goeman's global test p-value** | **Goeman's global test p-value** |
| Chondroitin sulfate biosynthesis | 0.0000005 | 0.0000293 |
| Prion disease | 0.0047368 | 0.0000402 |
| Type II diabetes mellitus | 0.0000009 | 0.0000637 |
| Adherens junction | 0.0001848 | 0.0000909 |
| Insulin signaling pathway | 0.0000215 | 0.0001255 |
| Colorectal cancer | 0.0000776 | 0.0001955 |
| Nicotinate and nicotinamide metabolism | 0.000017 | 0.0002499 |
| TGF-beta signaling pathway | 0.0001248 | 0.0002508 |
| Glycosphingolipid biosynthesis - ganglioseries | 0.0000888 | 0.0003341 |
| Glycan structures - biosynthesis 1 | 0.000001 | 0.0003474 |
| Glycosaminoglycan degradation | 0.0000219 | 0.0004338 |
| Regulation of actin cytoskeleton | 0.0001358 | 0.0004888 |
| Glycosphingolipid biosynthesis - globoseries | 0.0003157 | 0.0006576 |
| GnRH signaling pathway | 0.0000201 | 0.0006772 |
| Phosphatidylinositol signaling system | 0.0043828 | 0.0008082 |
| Focal adhesion | 0.0007751 | 0.0008178 |
| VEGF signaling pathway | 0.0001909 | 0.0008444 |
| ECM-receptor interaction | 0.0019389 | 0.0008649 |
| Gap junction | 0.0000441 | 0.000874 |
| Glycan structures - degradation | 0.0000461 | 0.000919 |
| Glycan structures - biosynthesis 2 | 0.0007425 | 0.0010205 |
| Wnt signaling pathway | 0.0000087 | 0.0010884 |
| Cell Communication | 0.0020388 | 0.0016939 |
| Calcium signaling pathway | 0.0000031 | 0.0021013 |
| Fc epsilon RI signaling pathway | 0.0003121 | 0.002286 |
| mTOR signaling pathway | 0.001165 | 0.002836 |
| Hedgehog signaling pathway | 0.0007788 | 0.0030842 |
